# Effects of Vibroacoustic Stimulation on Psychological, Physiological, and Cognitive Stress

**DOI:** 10.3390/s24185924

**Published:** 2024-09-12

**Authors:** Charlotte Fooks, Oliver Niebuhr

**Affiliations:** Centre for Industrial Electronics, University of Southern Denmark, 6400 Sønderborg, Denmark; olni@sdu.dk

**Keywords:** electroencephalography, electrocardiogram, heart rate variability, vibroacoustic technology, vibroacoustic sound massage, stress

## Abstract

Global stress is widespread in today’s post-pandemic world of political and economic uncertainty. Vibroacoustic technology is a vibrotactile intervention with multiple uses, but its impact on stress lacks interpretation. This research assessed if the vibroacoustic technology of a Vibroacoustic Sound Massage (VSM) can reduce psychological, physiological, and cognitive stress. The Perceived Stress Scale (PSS-10) and electrocardiogram (ECG) and electroencephalogram (EEG) biosignals were used to quantify results. Participants were divided into Low-Stress and High-Stress groups. The ECG results show VSM increased parasympathetic activity for all participants, with the Low-Stress group being more affected. The EEG results indicate increased concentration, reduced arousal, and increased relaxation, with participant well-being non-significantly affected, though variability in this metric was homogenised after VSM. Together, these results validate VSM as an effective support tool for stress management; however, further research is required.

## 1. Introduction

COVID-19 generated global stress and illuminated the scale of an existing mental health crisis. A meta-analysis conducted during the pandemic showed world stress prevalence at 36.5% with psychological distress at 50% [1]. The epidemic endorsed and supported the destigmatisation of mental health. Mindfulness knowledge dissemination rose together with spiritual practitioner popularity and a widespread prioritisation of self-care interventions. The post-pandemic period saw a surge in physical health and wellness spaces in the West, with holistic Eastern wellness practises (including meditation, breathing exercises, sound baths, and yoga) becoming commonplace in daily routines. This focus on mental health improvement spawned a booming wellness technology industry and exponential growth in new biotechnologies. The severed connection between brain and body once supported by Western science is now outdated, with scholastic knowledge shifting to bridge the gap [2,3,4].

Established well-being and stress management interventions include yoga [5,6,7], music [8,9], and meditation [10], with research linking their soothing effects to psychobiological vagal tone mediation [11,12,13]. Vagal tone describes the electrical activity of the vagus nerve, which extends from the brain stem, down the spinal cord to the abdomen, and connects to major organs including the heart, gut, and lungs. Heart rate variability (HRV) is a measure of vagal tone and indicates activity in the nervous system [14].

The central nervous system (CNS) includes the brain, spinal cord, and a large nerve network called the peripheral nervous system (PNS). The PNS communicates between the CNS and the body and is composed of the somatic and autonomic nervous systems. The somatic nervous system carries peripheral sensory information (such as indicating when something is too hot to touch), while the autonomic nervous system governs subconscious activities like digesting, breathing, and blood pressure [15,16].

With regard to physiological stress, the focus of the present study is the autonomic nervous system (ANS). This constitutes the enteric, sympathetic, and parasympathetic nervous systems. The enteric nervous system regulates the gut, the sympathetic nervous system (SNS) regulates a “fight or flight” response that is present when we are on high alert, and the parasympathetic nervous system (PNS) regulates a “rest and digest” response, occurring during periods of rest and calm.

**Figure 1:**
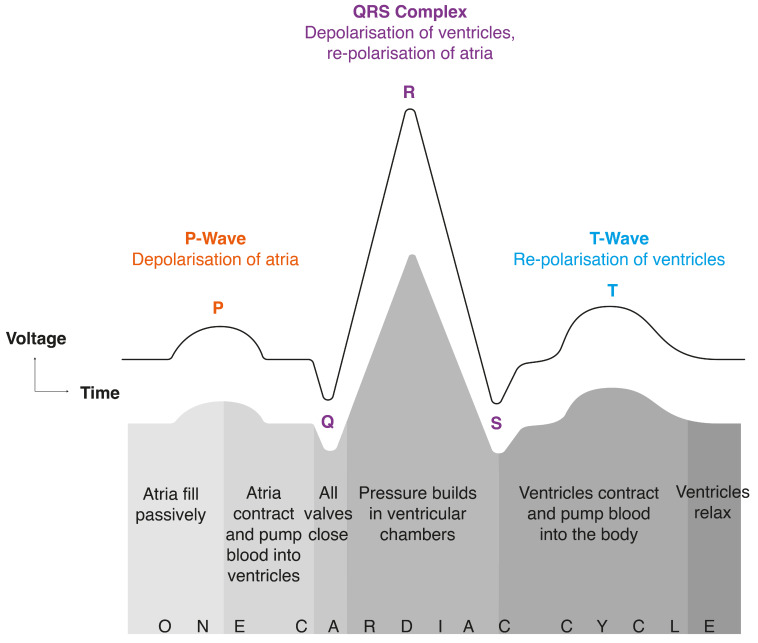
Description of a PQRST complex (and concurrent cardiac cycle) recorded by an electrocardiogram (ECG).

Vagal tone determines how stressed the nervous system is and can be measured with HRV using an electrocardiogram (ECG) [14]. ECG records sinus rhythm or the rhythm of the heart. The sinus node determines the rhythm by generating electrical signals that make the heartbeat. One cardiac cycle is called the PQRST complex. It is a process that repeats in healthy humans to make a heartbeat. Figure 1 itemises each stage of a PQRST complex.

**Figure 2:**
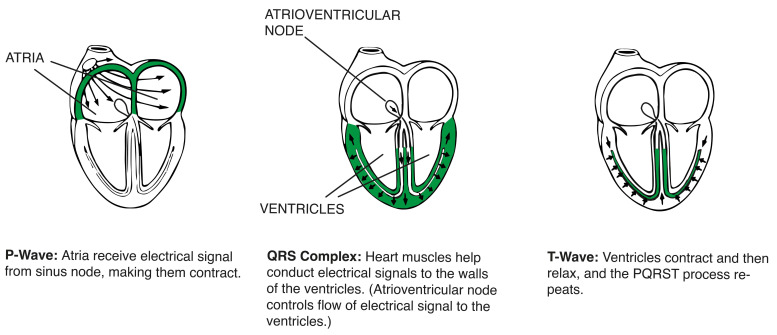
Movement of electrical signals sent by the sinus node during a PQRST complex (one cardiac cycle) [17].

Anatomically, electrical signals emitted by the sinus node make the heart atria contract. These signals then move through the atrioventricular node and cause the ventricles to contract, as shown in Figure 2.

**Figure 3:**
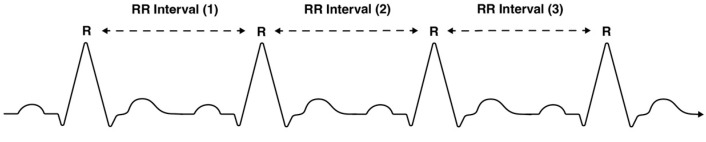
Visualisation of an ECG with RR intervals marked.

Electrical activity of the sinus node is defined by a balance of sympathetic nervous system activity (which accelerates it) and parasympathetic nervous system activity (which slows it). Fluctuations in blood pressure, heart rate, and vascular tone are all determined by the sympathetic and parasympathetic nervous systems. This is an autonomic nervous system (ANS) state termed sympathovagal balance, which is dictated by sympathetic and parasympathetic influences. HRV is a measure of sympathovagal balance—it is a metric of the differences between successive RR intervals (the time gap between R’s) in a PQRST complex. An RR interval is the difference between successive R waves in a PQRST complex (see Figure 3). Parasympathetic activity is indicated by a high HRV reading, while low HRV indicates sympathetic activity [18].

The present study assesses the effects of vibroacoustic technology on stress, measured using a subjective stress measure, i.e., the Perceived Stress Scale (PSS-10). In addition, direct, quantitative measurements are carried out based on the established ECG or HRV signals described above. This established biosignal source is further supplemented by electroencephalography (EEG) signals to measure cognitive stress in addition to the physiological stress captured by EEG/HRV signals. In accordance with their supporting role, these EEG signals and measurements are not described in the Introduction, but in the context of the methods described in Section 2.2.4.

### State of the Art

The term “vibroacoustic” refers to a bimodal approach in which people simultaneously receive tactile, and auditory stimulation; “vibroacoustic” stimulation is distinguished from “vibrotactile” stimulation, which only refers to the tactile low-frequency stimulation of the intervention. Conceptualised in the 1980s by therapist Olav Skille and music therapist Juliette Alvin, sinusoidal waves and/or music are played through specifically designed furniture with in-built speakers. Uses of the technology are multifaceted, including pain reduction [19,20,21,22,23], physiological recovery [24], and relief from psychological afflictions such as anxiety [25], depression [26], and insomnia [27].

Vibroacoustic research presently lacks methodological conformity, with extensive diversity in session duration (ranging from twenty minutes to one hour), recurrence (as individual or regular sessions), and sonic attributes (featuring repetitive low-frequency sinusoidal waves, soundscape, music, or a combination of these). Similarly diverse is the terminology, labelled as tactile low-frequency vibration, vibroacoustic therapy (VAT) [28], VA treatment [29,30], physioacoustic therapy (PAT) [31,32], the PA method [33], and vibroacoustic music (VAM) [34]. Consequently, the field remains unestablished. To wean out methodological inconsistencies, consideration should be taken with regard to standardising the following: exposure times, instrumental and bass frequencies, sonic elements (nature sounds, instrumentation, musical aspects, repetitive structures), and the level of emitted vibrational force. To facilitate this, more research needs to be conducted on the efficacy of each aspect. Naturally, aliments will be affected differently by each variable, and there will be diversity in outcomes—in the same way that different treatments exist for physiological and psychological complaints. Results from these studies would support methodological standardisation in future studies.

Preliminary research suggests that the intervention can relieve stress [35], though assessment of this is scarce [35,36,37,38]. To the authors’ knowledge, four studies to date have specifically compared the effect of vibroacoustic interventions on stress using biosignal measurements.

The first is a double-blinded randomised controlled trial of 54 university students by Kantor et al. (2022) [35], who compared an experimental group (VAT) to a control group (placebo). Both groups listened to the same piece of music developed for the study on a reclined wooden chair structure, the VIBROBED^®^. The VAT group was exposed to low-frequency sound (LFSs) within a frequency range of 0–100 Hz. Exposure lasted twenty minutes for both groups and participant heart rate variability (HRV) was assessed for an acute stress response. HRV results showed that, for the VAT group, exposure to LFS statistically increased parasympathetic nervous system activity. This indicated heightened nervous system relaxation, showing that VAT can be used as a stress management tool for university students.

The second study by Vilímek et al. (2022) [36] also used a VIBROBED^®^ as well as the VIBRATICS I vibroacoustic device. The latter is a padded flat vibrating surface upon which participants lay in the supine position. HRV was used to measure stress, in addition to a mood checklist (UWIST-MACL) and the PSS-10. The research assessed the effect of low-frequency vibration (LFV) on subjective stress perception and physiological function in 24 participants. The study design used only sounds of nature with no musical elements. Three conditions with varying sonic and tactile stimuli were tested, each lasting twenty minutes. The first included the natural sound of a forest river paired with a constant 40 Hz LFV without modulation; the second used the same nature sound and LFV with the addition of amplitude modulation; and the third condition was a placebo using the nature sound alone with neither LFV nor modulation. Both HRV and stress perception were insignificantly affected in all conditions. These results differ from other research using LFV in combination with music that assessed the effect of VAT on physiological functions [23,37,38,39,40,41]. This illustrates the necessity for a musical element during interventions. Additionally, larger studies are required to quantify significant results of LFV.

Delmastro et al. (2018) [37] conducted the third study that assessed the effects of a vibroacoustic intervention on stress using biosignals. Physiological impact on eight participants was measured using HRV, electrocardiogram (ECG), and the Galvanic Skin Response (GSR). Their study design is markedly comparable to the present research because of the implementation of ECG and HRV metrics. An AcusticA vibroacoustic chaise longue was assessed. This is a deep wooden chair supporting the head, body, and legs. All participants were exposed to an ill-defined twenty-minute VAT therapy “melody” within the frequency range of 20–17 kHz, constituting “specific music and melodies composed by a music therapist” [37]. The ECG and GSR results showed VAT-induced physiological relaxation, with participants also reporting muscular relaxation. With respect to the study’s sample size, a far greater participant pool is required to cater for inter-individual differences.

The fourth article is a proposal for a scoping review of the physiological effects of VAT, authored by Kantor et al. (2022) [42]. The inclusion criteria were broad, accommodating all studies using physiological biomarkers of stress. Because of these broad inclusion criteria, the results are likely to deviate greatly. The paper proposed an analysis of the effects of vibroacoustic technology (sedative or stimulatory), in addition to the acknowledgement of the most efficacious sonic attributes for vibroacoustic stress management. Unfortunately, the final scoping review to which Kantor et al.’s (2022) protocol points does not exist. A scoping review by the same author exploring the effects of vibroacoustic therapy on adult pain [28] collated 430 records and concluded that the research remains too sparse for accurate identification of specific VAT pain management characteristics. Despite this finding, a scoping review defining vibroacoustic intervention stress relieving characteristics would be markedly beneficial for the field and further hardware development. As evidenced, vibroacoustic technology requires extensive exploration.

Vibroacoustic Sound Massage (VSM) is used throughout the present paper to refer to the VibroAcoustics Bass Module [43] that is assessed. This research uses biosignal measurements to analyse the effects of VSM on human stress (quantified as psychological, physiological, and cognitive stress). EEG and ECG biosignals are recorded before, during, and after exposure. PSS-10 scores recorded before and after exposure are used as an intra-participant control baseline for High-Stress or Low-Stress groupings. All metrics are collated to assess the accurate multimodal effects of VSM on physiological, psychological, and cognitive stress. The existing literature lacks an assessment of concurrent neural, cardiovascular, and cognitive stress before, during, and after VSM exposure.

The present study fills gaps in the current vibroacoustic research in multiple ways. First, this study assesses the effect of VSM on stress using concurrent EEG and ECG biosignal metrics, in addition to a subjective stress measure the PSS-10. Secondly, it is conducted using a vibrotactile modality on which participants lay fully flat to facilitate maximised contact. Thirdly, this research is conducted on a larger participant sample (38 participants) as compared with studies of smaller sizes. Fourthly, the VSM sound design features quantified stress-reducing sonic characteristics (including rhythmicity and pitch, as further discussed). Novel in its approach, this research assesses if supine-position VSM is efficacious as a stress management tool, comprehensively quantified with EEG, ECG, and PSS-10 measures. To the authors’ knowledge, these stress metrics have never been concurrently recorded, collated, and analysed in one vibroacoustic research methodology before.

This study poses three research questions (RQs) and associated hypotheses (Hs):

Research Question One (RQ1) on *cognitive stress* is as follows: How does VSM affect mental processing?

**Hypothesis** **1** **(H1).** *VSM positively affects concentration (theta/beta ratio), relaxation (beta/alpha ratio), and well-being (frontal/alpha asymmetry)*.

Research Question Two (RQ2) on *physiological stress* is as follows: How does VSM affect the autonomic nervous system, vagal tone and heart rate?

**Hypothesis** **2** **(H2).** *VSM increases HRV, causing more parasympathetic activity (increased RMSSD and HF) and less sympathetic activity (reduced SDNN and LF). VSM will reduce HR BPM [37]*.

Research Question Three (RQ3) on *psychological stress* is as follows: Are participants with higher PSS-10 scores more impacted by VSM?

**Hypothesis** **3** **(H3).** *VSM benefits individuals with higher perceived psychological stress [35]*.

In the following sections, the apparatus, methodology, and study design are clearly explained, the results are analysed and discussed, and conclusions are drawn.

## 2. Materials and Methods

Vibration is integral to human functioning. Omnipresent, it determines everything from heart contractions to nerve impulses and the resonant frequency of groups of cells. Resonant frequencies are naturally occurring vibrations in the body, which can synchronise with external sonic vibrations. Liquid, tissue, and bone all have different resonant frequencies. Sonic tactile vibrations emitted by vibroacoustic technology are designed to affect resonant frequencies inside the body. Single sine waves can activate areas that share a resonant frequency, while more complex tones (like those in music) cause the resonance of multiple areas simultaneously [44,45]. Vibroacoustic technology facilitates a tactile reception of sonic vibration. It uses low-frequency sine waves and/or music within the range of human hearing to provide an embodied or haptic experience of sound.

The internal mechanisms supporting vibroacoustic efficacy are yet to be fully understood (as outlined in Section State of the Art). Thus, the present study broadens knowledge by using biosignals to measure the effects of VSM on cognitive, physiological, and psychological stress. To quantify cognitive stress, brainwaves were recorded using an electroencephalogram. EEG records electrical activity in the brain, which can be used to deduce mental disposition as one of the following five brain wave states: delta (0.5–4 Hz), theta (4–7 Hz), alpha (8–12 Hz), beta (16–31 Hz), and gamma (36–90 Hz) [46]. High-frequency brain waves are associated with greater cognitive arousal, while low-frequency brain waves indicate a calmer mental state. Muse S (Gen 2) is a portable, comfortable, and clinically accurate EEG device used in this study to map prefrontal and temporal lobe activities accurately.

To measure physiological stress, an electrocardiogram is used to record electrical activity in the heart. This electrical activity changes relative to how stressed the nervous system is. ECG records electrical impulses of the heart through heart rate (HR) beats-per-minute (BPM) and heart rate variability (HRV). Measured using HRV, the vagus nerve and vagal circuit are crucial for emotion regulation and the stress response [47]. Mediated by the central nervous system, the vagus nerve is the primary mechanism for heart–brain interactions and transmits sensory information between the two. It is integral for controlling heart rate, digestion, blood glucose, and other visceral functions [48]. The primary function of the vagus nerve is to sustain homeostasis. Vagal tone is indicative of vagus nerve functioning, with increased vagal tone representing parasympathetic activity (a relaxed response). HRV is used to measure vagal tone and the status of the vagus nerve. In the present study, a Polar H10 Heart Rate Monitor chest strap [49,50,51] was used to measure ECG data accurately from the chest region, which recorded HR BPM and HRV. ECG readings for a calm unstressed nervous system would show low HR BPM and high HRV, the latter illustrative of parasympathetic nervous system activity. On the contrary, a stressed nervous system would show high HR BPM and low HRV, indicative of activity in the sympathetic nervous system.

Vagus Nerve Stimulation (VNS) is a clinical neuromodulation practice used to modulate and increase vagal tone. VNS can provide therapeutic relief from a wealth of conditions including epileptic seizures [52,53], low mood and depression [54,55], obesity [56], and migraines [57]. VNS often involves an invasive medical implant, while alternative non-invasive nervous system treatments use surface electrodes, music and vibration, and ultrasound [58,59]. Vibroacoustic interventions are neurophysiologically beneficial, and this research posits that vibroacoustic technology could be used as an alternative non-invasive solution to VNS for stress regulation.

Psychological stress is assessed in the present study using the Perceived Stress Scale (PSS-10) [60] questionnaire to record changes in perceived stress before and after exposure. The PSS-10 is a 10-item self-report questionnaire and a widely used diagnostic support tool. PSS-10 scores are positively correlated with stress (higher scores indicate higher stress). Using the average participant score as the dividing threshold, the participants were categorised into a High- or Low-Stress group by their initial PSS-10 score. Psychological stress was quantified by comparing questionnaire responses before and after the vibroacoustic intervention.

This research adopted a within-subjects mixed-methods approach, with quantitative (PSS-10, EEG and ECG) and qualitative (verbal first-person accounts) data streams analysed and correlated.

### 2.1. Apparatus

#### 2.1.1. EEG Apparatus: Muse S (Gen 2)

**Figure 4:**
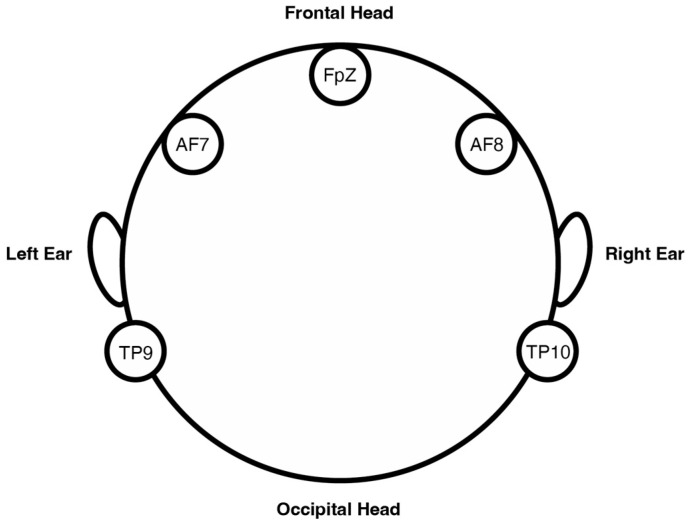
Locations of nodes in the Muse S (Gen 2) electroencephalogram (EEG) headband.

**Figure 5:**
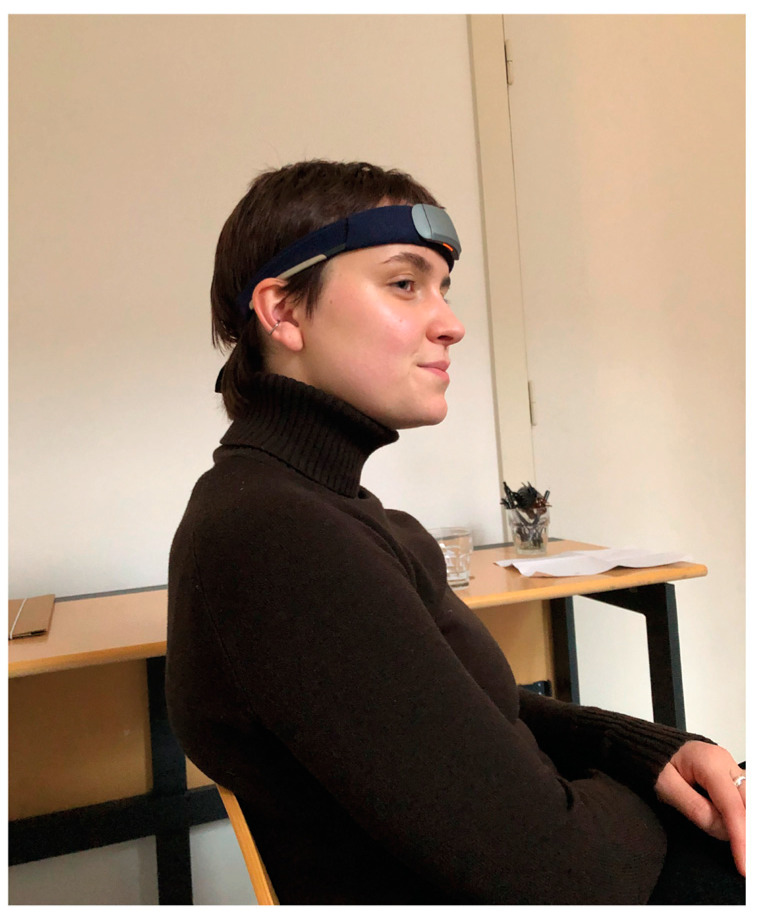
A participant wearing a Muse S (Gen 2) EEG headband.

A Muse S (Gen 2) EEG device was used to measure the electrical activity of the delta (δ), theta (θ), alpha (α), beta (β), and gamma (γ) frequency bands. It has one reference electrode and six channels, as well as four working nodes and two auxiliary channels. Four nodes measure electrical current with respect to a zero-voltage reference node. Two auxiliary channels are used for calibration, which can record additional peripheral signals (the latter was unnecessary for the purpose of the present study). The four working electrodes are located at AF7, AF8, TP9, and TP10, with a single reference electrode at FpZ. AF7 and AF8 measure activity in the frontal lobe, while TP9 and TP10, located above the ears, record information from the temporal lobes (see Figure 4 and Figure 5). AF stands for “anterior frontal” and TP for “temporal parietal”, i.e., two brain areas associated with the prefrontal cortex and the temporoparietal junction, respectively. These areas of the brain are responsible for decision-making, attention, and sensory integration, i.e., aspects of cognitive processing that, amongst other things, relate to the emotional processing and evaluation of stimuli; see, for example, [61]. 

Accordingly, Muse S was chosen for the present study not only because of its reliability known from previous papers but also because its measurements concern exactly those aspects of perception that are at the centre of the research questions. Moreover, the Muse S (Gen 2) headset offered the necessary precision and mobility and, last but not least, the integration of the sensors into a soft headband provided the comfort participants needed when lying still for 45 min.

**Figure 6:**
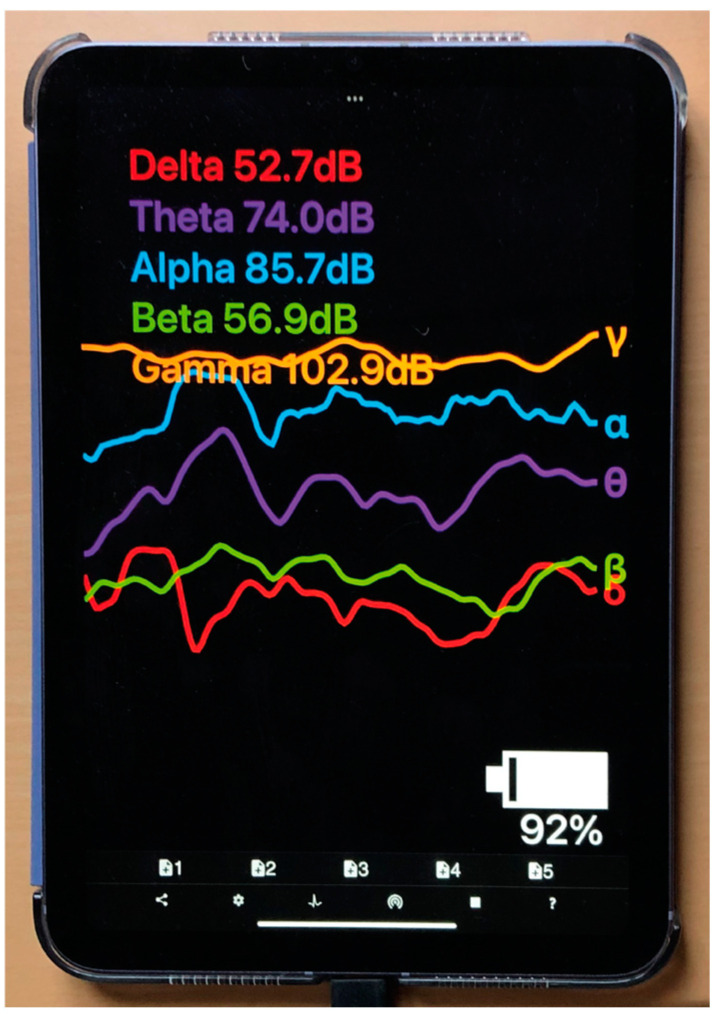
Mind Monitor iPad application displaying live EEG data.

To avoid measurement errors and ensure sustained electrode contact, the participants remained still throughout the duration of VSM exposure. This EEG device recorded raw data from the 4 electrodes at a sampling rate of 256 Hz. It was connected via Bluetooth to the iPad application Mind Monitor [62], which records signals from the headband in real time. Mind Monitor processed the raw EEG data from each of the four electrodes and performed an FFT spectral analysis. That is, like light, the EEG signal consists of a multitude of wavelengths or frequencies. The spectral analysis broke the overall signal down into these frequency components and divided them into the following five frequency bands: delta (<4 Hz), theta (4–7 Hz), alpha (8–15 Hz), beta (16–31 Hz), and gamma (>31 Hz) [63]. These were visualised in the app. See Figure 6 as well as Section 2.2.3 and Section 2.2.4 for further details.

This research assessed the effects of the intervention on EEG using three dependent variables of cognitive stress. Concentration was measured using the theta/beta ratio (TBR) [64]; arousal was measured with the beta/alpha ratio (BAR) [65]; and well-being was assessed using frontal/alpha asymmetry (FAA) [66]. All parameters are discussed at length in Section 2.2.4.

#### 2.1.2. ECG Apparatus: Polar H10 Heart Rate Monitor

**Figure 7:**
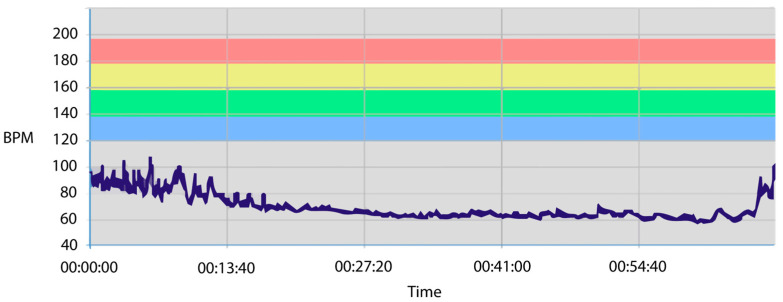
A participant’s ECG recorded during this study. The horizontal axis shows beat-per-minute (BPM) values. Colours refer to neutral (grey), good, slightly too high, and much too high (red) heart rate values (beats per minute, bpm) during physical exercise.

A Polar H10 chest strap was used to measure ECG as it offers optimal accuracy and smartphone connectivity [49]. The Polar H10 chest strap uses a sampling rate of 1000 Hz, and it is the gold standard for RR interval assessments [50] used by professional athletes and in research to calculate HR and HRV values automatically. This device was favoured because of its reliability and validity with respect to recording HRV parameters accurately [51]. The apparatus was specifically chosen for these reasons, with the electrode dampened prior to application to mitigate potential measurement errors through loss of sensor contact. The device was connected via Bluetooth to the smartphone recording app Heart Rate Monitor [67]. Figure 7 is an ECG recording of a participant during this study.

#### 2.1.3. Questionnaire: Perceived Stress Scale PSS-10

The Perceived Stress Scale (PSS-10), developed by Cohen et al. (1983) [60], is a widely used 10-item questionnaire designed to evaluate stress levels in individuals. It assesses the extent to which individuals perceive their lives as unpredictable, uncontrollable, and overwhelming. It can be completed in five minutes, with respondents rating the frequency of their feelings on a Likert scale from 0 (“never”) to 5 (“very often”). All questions are formulated so that higher values indicate a higher level of perceived psychological stress. The final score is then derived by summing responses across all items, with higher scores indicating elevated levels of perceived stress. The maximum score is 50 and the minimum is 0.

The PSS-10 has been validated across adolescent and adult populations over the past 40 years. Lee’s (2012) [68] meta-analysis highlighted the internal consistency in the questionnaire, revealing that Cronbach’s alpha values (a measure of internal reliability) exceeded >0.70 across 11 of the 12 studies available at that time. More recent empirical evidence shows it can be used in meaningful comparisons across ethnic and linguistic groups. The work by Baik et al. (2019) [69] demonstrates its effectiveness in facilitating stress-related comparisons between English- and Spanish-speaking Americans. Normative data are also available for a number of countries. Denmark is not yet included, though its neighbours Germany [70] and Sweden are [71]. A validated Danish version of the questionnaire was created by Eskildsen et al. (2015) [72]. The present study used the original English version.

#### 2.1.4. Vibroacoustic Apparatus: Device and Audio

**Figure 8:**
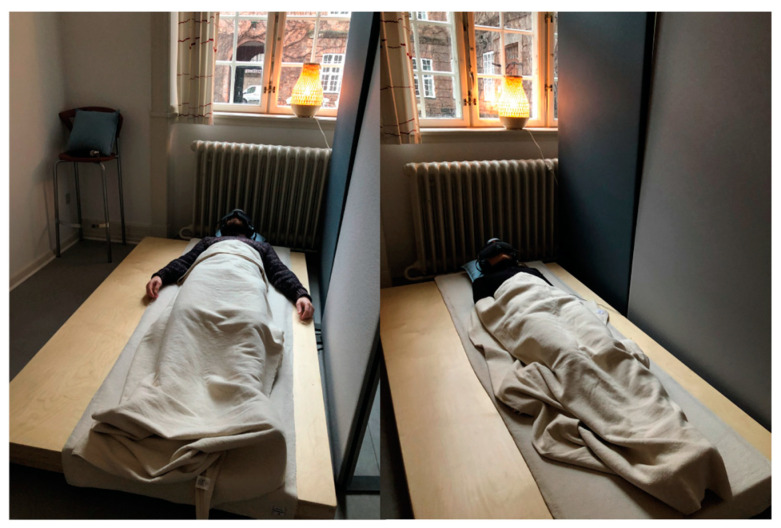
Participants in the research setting experiencing a VibroAcoustics Sound Massage (VSM).

This study tests the efficacy of a VibroAcoustics Bass Module [43] on which participants lay in the supine position. The wooden device is the size of a single mattress and houses three transducer speakers with a frequency range between 20 and 80 Hz. A mobile phone was connected via aux to the module, which played sound designed for this study. The participants listened to this through Bose QuietComfort 35 II noise-cancelling headphones (see Figure 8).

A 45 min soundscape was specifically designed with a restorative cognitive effect that featured live recordings of forest sounds, wind, water, rainfall, and birdsongs [73,74,75]. The isochronic tones (single tones of the same frequency at repeated intervals) occurring in these natural sounds improve cognitive wellness [76] and increase parasympathetic activity [77]. Field recordings from Denmark, Portugal, Spain, Israel, and India featured mechanical industrial rhythms and Eastern instrumentation with a repetitive drone-like quality. These nature sounds and rhythms were heavily reverberated in postproduction and mixed together with electronic harmonies. The soundscape assimilated learnings from brain entrainment research that documents the cognitive effect of repetitive rhythmic sonic structures [78,79,80,81]. Integral for vibrotactile stimulation, the soundscape also featured 20–80 Hz bass frequencies. These were of alternating tones with a repetitive rhythmic nature. The “ideal” frequency for vibroacoustic stimulation remains contested, though 40 Hz is postulated to be the most efficacious [82]. Either as a continuous sinusoidal wave or enmeshed into other sounds as in the present study’s soundscape, 40 Hz consistently demonstrates the most advantageous physiological outcomes. Reasons for this are manifold and include the following: as transducers are less efficient at a lower range, low-frequency vibrotactile sound must be greater than 27 Hz to be physically perceived [82]; it may also be because brainwave entrainment occurs best at 40 Hz, which is the lower end of the frequency range at which the gamma band oscillates [28,83]. This is because gamma brainwaves have the widest range, connecting both hemispheres and lobes. These oscillations are associated with consciousness, memory, emotion, heightened arousal, concentration, and sensory integration [84].

### 2.2. Method

#### 2.2.1. Recruitment

The participants were recruited through social media advertisements. The exclusion criteria included the following: prior VSM experience, pre-existing mental health conditions, and/or chronic pain. This study was single-blinded to reduce participant priming and thus biases in the results.

A random sample was selected from the applicants to participate in this study. A total of 40 participants voluntarily took part, including 13 males and 27 females between the ages of 20 and 35 years old, with a mean age of 28. Two participant data sets were excluded from the analysis because of premature study termination.

#### 2.2.2. Experimental Procedure

This research was conducted at the National Institute of Public Health in Copenhagen, Denmark. The study setting was prepared with sound-absorbing mobile wall panels and designed as a calm environment with soft light and furnishings. Participants wore the Polar H10 chest strap and Muse S (Gen 2) headset at all times throughout this study.

This study consisted of the following 6 stages: normal activity (NA), prosody 1 (P1), rest state (RS), Vibroacoustic Sound Massage (VSM), Post-Vibroacoustic Sound Massage (PVSM), and prosody 2 (P2) (see Table 1). During stages P1 and P2, the participants read a text aloud. Both stages assessed speech prosody and are neither discussed nor analysed in the present paper. Results from these stages can be found in Fooks & Niebuhr (2024) [85].

To avoid priming biases, the requirements of each study stage were explained to the participants only before each stage started. There was one exception to this to ensure data validity, which was between the VSM and PVSM stages. Before VSM, participants were instructed to continue laying on the module with their eyes closed afterwards for five minutes, i.e., the duration of PVSM. PVSM measured EEG and ECG fluctuations immediately after VSM exposure; thus, distracting participants before this stage would have reduced result validity and increased noise in the data. Throughout data collection, live transcriptions were recorded by the researcher. Total study time was approximately 70 min to accommodate a time buffer for preparation and questions. After stage 6, the participants were invited to verbally share their experiences, and the results are itemised in Section 3.1.4.

#### 2.2.3. Processing of Biosignals

The biosignal data that were collected with the two apps went through a series of consecutive signal processing and enhancement steps, which, firstly, filtered out basic measurement errors and interferences from the raw signals and, secondly, converted the raw signals into meaningful parameters and variables. The corresponding processes happened as part of the respective apps, i.e., Mind Monitor [62] and Heart Rate Monitor [67].

To assess the cognitive impact of VSM, the summed power of specific frequency bands provided by the Mind Monitor app was analysed and compared (θ, α, β). Beforehand, the raw signals went through a high-pass filter to remove low-frequency drift, a low-pass filter to eliminate high-frequency noise caused by, e.g., the electronic components and the Bluetooth transmission, and a notch filter to remove power supply-induced signal noise (such as the typical 50/60 Hz buzz). In a subsequent step, a Fast Fourier Transformation (FFT with 512 points) was performed to break up the signal into its individual spectral frequency components (and their amplitudes). Finally, within each target frequency band (i.e., alpha, beta, and theta; see Section 2.1.1), the signal energy was integrated using the RMS (root-mean-square) procedure and converted into dB values. These dB values represented our measurements. In addition, the plausibility of these measurements were manually checked, with zero values and obvious outliers removed, as determined statistically by means of Q tests. Also, noisy data elements such as those around eye blinks and jaw clenches recorded by Muse S (Gen 2) were extracted before the data were analysed. Furthermore, note that the final measurements were taken per second. This means that they were mean values that integrated all dB values over the time window of one second (recall that the device’s sampling rate was 256 Hz). This further reduced the influence that unreliable individual values had on the final measurements.

Heart Rate Monitor’s measurements are based on blood volume change, determined by means of a camera or light sensor. That is, as the heart pumps blood, the blood volume in the capillaries changes with each heartbeat. These changes affected the amount of light that was absorbed or reflected and could be measured by the light sensor inside the belt system worn by the participants. The signal filtering and post-processing steps that were executed on the raw signal data were overall similar to those of Mind Monitor, as the framework conditions were also similar, i.e., a noisy raw signal and the additional noise sources related to power supply and Bluetooth transmission. So, like for the raw EEG signals, the raw heart rate (or light) signals were subjected to a low-pass filter to remove high-frequency noise caused by signal processing and transmission, a high-pass filter to eliminate low-frequency drift, and a band-pass filter tailored to the frequency range of interest for heartbeats, i.e., 0.5 to 3 Hz. The filtered signal was then analysed to extract the waveform corresponding to the pulse. Specifically, a peak detection algorithm (Pan–Tompkins) was applied to identify those peaks in the waveform that corresponded to individual heartbeats. HRV measurements were also taken based on that peak detection procedure. Potential artefacts and non-sinus events were cleaned from the data prior to analysis, i.e., RR interval outliers, ectopic beat, and arrhythmic events were removed. All further, subsequent manual checks and outlier removals were identical to those applied to the Mind-Monitor signals.

#### 2.2.4. Additional Explanations of the EEG Measures

Compared with the measures derived from the heart rate signals, which refer to time intervals and their degree of repetition and consistency, the EEG measures are more complex and, therefore, are separately explained below and classified with regard to their reliability and history.

Frontal alpha asymmetry (FAA) has been a valuable tool in psychological and neuroscience research for several decades. The measure is largely due to Davidson et al. (1985) [86], who explored the association between frontal EEG asymmetry and affective style. Their results demonstrated that greater left frontal activation was associated with positive affect and approach behaviours, while greater right frontal activation was associated with negative affect and withdrawal behaviours. Since then, FAA has provided important insights into the neural correlates of emotion and motivation and has been applied in both research and clinical settings. It has become an effective and reliable measure of emotional valence, i.e., the positive vs. negative assessment of a situation or stimulus. For example, Stewart et al. (2010) [87] found that FAA could predict depression relapse with approximately 75% accuracy. It was adopted here according to the established formula, i.e., FAA = log(alpha power at left electrode) − log(alpha power at right electrode), with the only exception being that instead of AF3 and AF4, the more frontal and lateral AF7 and AF8 electrodes were used. However, this selection may have made our measurements and analyses more conservative, cf. Alyan et al. (2021) [88]. In the context of the present study, FAA data are interpreted in terms of the degree of the participants’ well-being.

The beta/alpha ratio (BAR) is another well-established measure in EEG research that has been used since at least the late 20th century. Studies like that by Pivik & Harman (1995) [89] investigated the changes in alpha and beta EEG components under varying mental workloads, in this way, establishing a basis for understanding how these frequencies reflect different cognitive states. In the last decade, BAR has become a widely used measure of emotional regulation and arousal. Authors such as Kislov et al. (2022) [90] refer to BAR as an “engagement index”. In their study, this measure was “particularly predictive for the aggregated effect of advertising” (p. 9). Note that the BAR measure is fundamentally different from FAA in that it does not reflect valence but arousal. Thus, in the present study, BAR was used to measure the level of relaxation of the participants.

The third measure in the present study was the theta/beta ratio (TBR). Studies have shown that the theta/beta ratio can be an insightful measure of attentional processes. The TBR is particularly noted for its application in ADHD diagnosis and treatment but, more generally, indicates a person’s level of concentration or focus. For example, Swartwood et al. (2003) [91] found that the TBR could distinguish children with ADHD from children without ADHD with an accuracy of about 85%. Twenty years earlier, a study on cerebral ischemia by Köpruner et al. (1984) [92] highlighted that “one of the most remarkable results [...] was that the quotient theta/beta power showed the highest contribution to the overall discriminative power” of the multiparametric score they used (p. 46). Van Son et al. (2019) [93] found the TBR to be effective in identifying and quantifying the mind-wandering episodes of people, and this is also the background of its application in the present study. The TBR was used to determine how many participants let their thoughts run free and wander under VSM.

#### 2.2.5. Statistical Analysis

The statistical analysis of our measurements was based on analyses of variance and was carried out according to the same principles across all measures. Each measure represented the dependent variable of its respective statistical test. The six exposure/stimulus conditions of the experiment were tested, i.e., NA, P1, RS, VSM, PVSM, and P2 (see Table 1), leading to statistically above-chance differences in the level of the measured values of the dependent variable. An alpha error level of *p* ≤ 0.05 (the error of falsely attesting a significant difference) was considered above chance and, therefore, statistically significant. The six conditions are summarised under the independent variable stage. Potential gender differences in response to VSM were not separately considered in our statistical analysis. The sample size was too small split into independently analysed subsamples of male and female participants. Although this is a limitation (as addressed in Section 4.5), the focus of the present study was not on participant-specific VSM effects but on the participants’ lifestyles and how they relate to stress and well-being in connection with VSM stimulation. Moreover, the existing literature does not contain particularly strong evidence that gender is a necessarily relevant factor in EEG or heart rate biosignals. For example, a meta-analysis by Van Der Vinne [94] found no gender differences in FAA measurements or their predictive power for depression, except for people older than 53 years (which was older than any of the participants in the present study). In addition, a large-scale study on the standardisation of HRV measurements [95] emphasized that “a lack of sex differences and higher or even lower HRV in women have been reported” (p. 99).

Before each analysis, the measurements were checked for their variance homogeneity, i.e., whether the values in each of the six levels of the independent variable stage had a similar spread. If this was the case, a parametric, univariate ANOVA with repeated-measures design was run (all participants went through all six conditions). If there was no variance homogeneity, the non-parametric counterpart of the univariate, repeated-measures ANOVA was used, i.e., the Friedman test. The implications of the presence and absence of variance homogeneity for the interpretation of the data are addressed in the Discussion Section.

The analysis was completed by post hoc multiple paired comparison tests with conservative Bonferroni corrections of alpha error levels. Depending on the type of analysis of variance (parametric vs. non-parametric), the paired comparisons were carried out either as paired t-tests or as Wilcoxon tests.

In the following, the results of each measure (dependent variable) are addressed individually. Each results summary is accompanied by two figures, i.e., a box plot and a hexagon plot. The box plot is based on the established quartile structure. Outliers are separately marked as unfilled circles and defined as values that are outside the 3rd quartile (75% of the distribution of the data) and additionally exceed the interquartile range by a factor of 1.5. The hexagon plot gives a quick overview of the number and nature of the significant differences in the post hoc multiple paired comparisons tests. Each of the six conditions of stage is shown with its mean, and thick black lines indicate the significant findings—in the form of solid lines when they are related to the VSM condition.

## 3. Results

### 3.1. Cognitive Stress (EEG)

Being negatively correlated with concentration, when the TBR is low, individuals are concentrated. A higher TBR indicates mind-wondering or unfocused thought [93]. The BAR is positively correlated with arousal. That is, high BAR values indicate that individuals are more alert, while lower scores demonstrate less experienced arousal and more relaxation [96]. Finally, frontal alpha asymmetry (FAA) is positively correlated with well-being. Thus, when greater well-being is experienced, FAA increases [66].

#### 3.1.1. Theta/Beta Ratio (TBR)—Concentration and Focus

**Figure 9:**
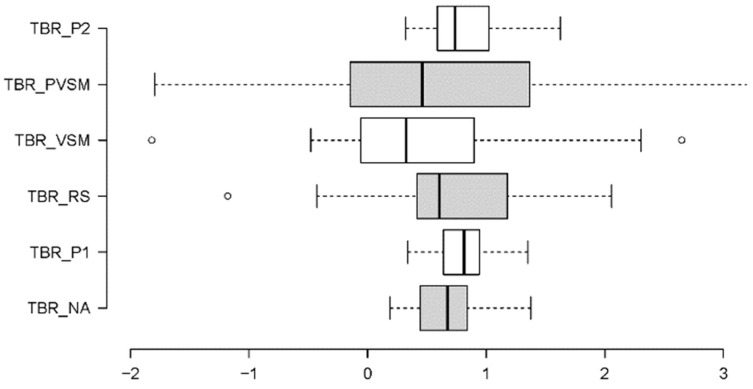
Boxplot summary of the TBR results for the six stages of this study, shown in chronological order from bottom to top. The horizontal axis shows dB ratios.

Because of heterogeneous variances, the Friedman test was applied to the TBR data. The overall effect size of this test was estimated in terms of Kendall’s W. The Friedman test revealed a significant main effect of stage on the average TBR levels across all participants (c^2^[5] = 14.714, *p* = 0.012, W = 0.1); see Figure 9.

**Figure 10:**
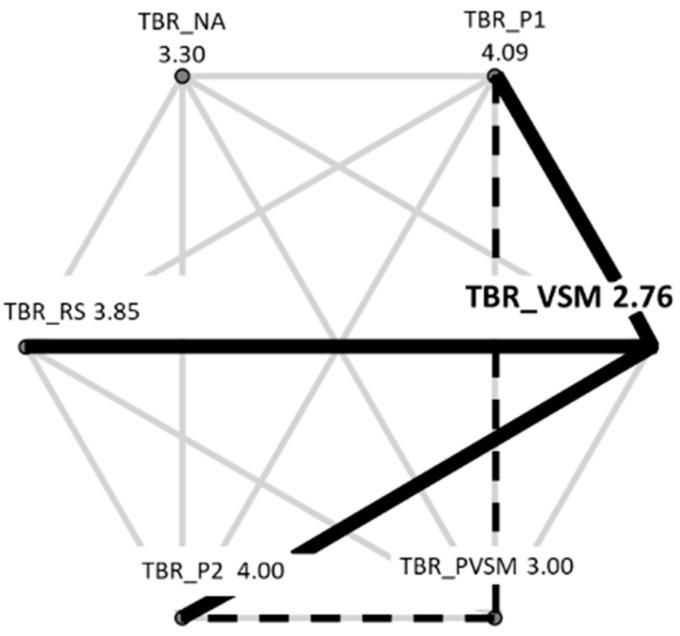
Overview of the Wilcoxon TBR test results. Thick black lines show significant differences between VSM and other stages (P1, RS, P2); dotted black lines indicate other significant TBR differences among stages (P1, PVSM, P2).

Additional paired comparisons in the form of Wilcoxon tests (with Bonferroni correction for multiple testing and r as an effective size indicator) showed that VSM (stage 4) differed significantly from stages P1 (stage 2) (W = 2.895, *p* = 0.004, r = 0.47), RS (stage 3) (W = 2.369, *p* = 0.018, r = 0.38), and P2 (stage 6) (W = −2.689, *p* = 0.007, r = 0.44); see Figure 10. More specifically, the participants’ TBR levels were lower during VSM stimulation (indicating higher concentration), compared with the resting stage (RS) that preceded the VSM, and the two text readings (P1 and P2). Post-VSM (PVSM) differed from the subsequent P2 stage, as it was not characterised by a significant increase in TBR levels relative to the VSM stage.

#### 3.1.2. Beta/Alpha Ratio (BAR)—Arousal

**Figure 11:**
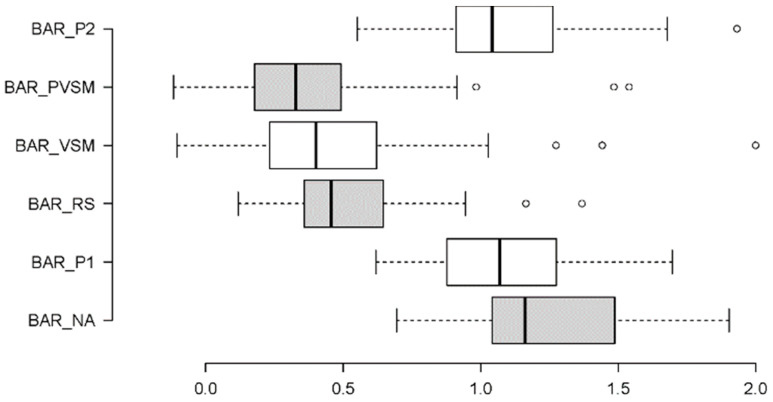
Boxplot summary of BAR results for the six stages of this study, shown in chronological order from bottom to top. The horizontal axis shows dB ratios.

A one-way repeated-measures ANOVA was conducted using the BAR measurements. As shown in Figure 11, there were strong differences among BAR levels in each stage. The results show a successive decrease in the participants’ BAR levels from the initial NA stage to the two stimulation-related stages VSM and PVSM. From PVSM to P2, the BAR again increased to almost the same level as in the initial NA stage; see Figure 11. Accordingly, the RM-ANOVA yielded a significant main effect of stage on the BAR measurements (F [5,160] = 29.588, *p* < 0.001, η_p_^2^ = 0.480).

**Figure 12:**
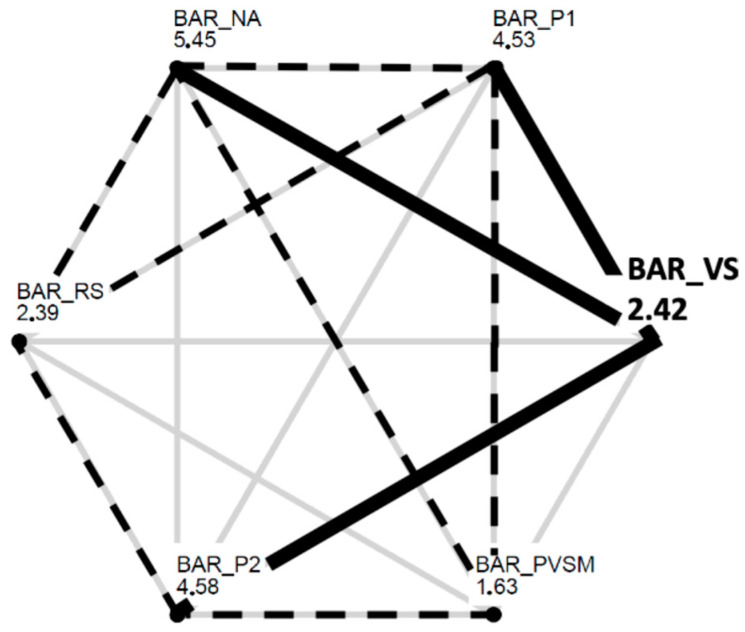
Overview of the paired-comparisons *t*-test results. Thick black lines show significant differences between VSM and other stages (NA, P1, P2); dotted black lines indicate other significant BAR differences among stages.

Multiple paired comparisons of the BAR levels among stages (with Bonferroni correction for multiple testing and Cohen’s d as a measure of effect size) revealed that VSM (stage 4) differed significantly from the P1 (t[37] = −7.161, *p* < 0.001, d = 0.53) and P2 (t[37] = −6.826, *p* < 0.001, d = 0.56) reading stages. The VSM stage was associated with considerably lower BAR levels than P1 and P2, which indicates less arousal, as illustrated in Figure 12. The same was true for the initial NA stage (t[37] = −9.160, *p* < 0.001, d = 0.51). The BAR level during VSM stimulation was significantly lower than that at the beginning of the experimental session during stage 1 (NA). In contrast, the BAR level during the two resting stages, the RS and PVSM, did not differ from the VSM stage. That is, the BAR levels remained low in both the stage preceding (RS) and following (PVSM) the VSM stimulation.

#### 3.1.3. Frontal/Alpha Asymmetry (FAA)—Well-Being

**Figure 13:**
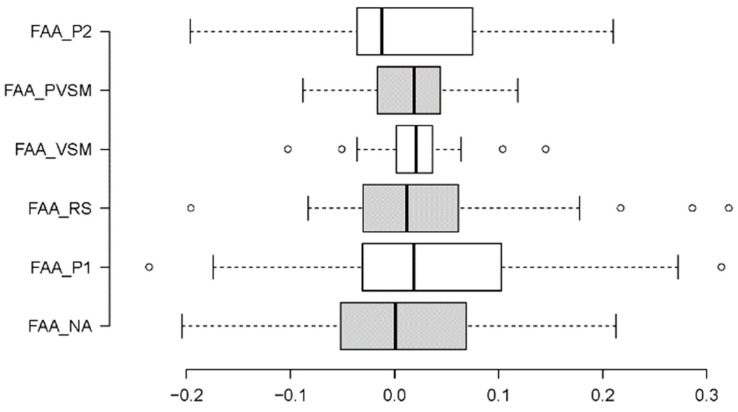
Boxplot summary of FAA results for the six stages of this study, shown in chronological order from bottom to top. The horizontal axis shows dB values.

A Friedman test was conducted to analyse the FAA data. As illustrated in Figure 13, the FAA measurements differed among the stages more in terms of their variations than in terms of their mean levels. Accordingly, the Friedman test was non-significant. Despite this, the VSM stage still stood out against the other stages in terms of its low variation in FAA measurements. A series of F-tests confirmed that FAA variance across participants was significantly lower during the VSM stage than during any other stage of the experimental session (with significance levels of at least F [37,14] = 0.111, *p* < 0.001).

#### 3.1.4. Qualitative Data: First-Person Verbal Accounts

To connect EEG data to first-person verbal accounts, participant responses were transcribed immediately after the experimental procedure. The results are itemised in Table 2.

The TBR results are consistent with descriptions of cognitive entrainment, concentration, and focus. The BAR results are consistent with verbal accounts of cognitive arousal and relaxed disassociation, the latter being regularly likened to a psychedelic experience. Although EEG FAA levels did not differ significantly among stages, reduced variability in this metric is illustrated by verbal accounts of experienced themes such as safety, security, and comfort.

### 3.2. Physiological Stress (ECG)

#### 3.2.1. Mean HR

**Figure 14:**
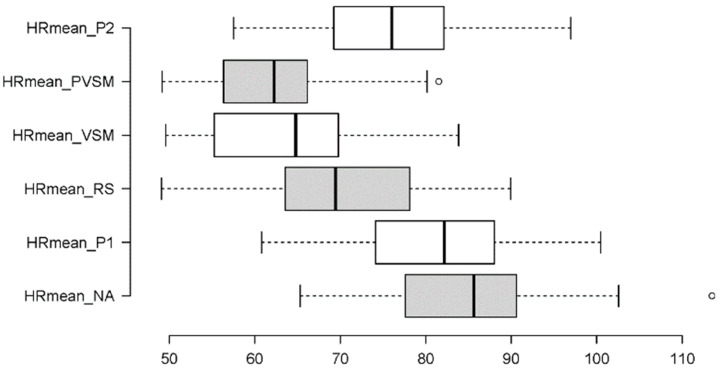
Boxplot summary of mean HR results for the six stages of this study, shown in chronological order from bottom to top. The horizontal axis shows beat-per-minute (BPM) values.

The distribution and variance characteristics of the data allowed us to analyse mean HR values using a one-way repeated-measures ANOVA. The main effect of stage on the participants’ heart rate levels was significant (F [5,160] = 4.232, *p* < 0.001, η_p_^2^ = 0.120). Figure 14 shows that the participants’ heart rate levels decreased successively (by about 15–20%) from around 80–90 BPM during the NA (stage 1) and P1 (stage 2) stages to around 70 BPM in the RS (stage 3). From here, the mean HR dropped further to between 60 and 65 BPM during the VSM (stage 4) and PVSM (stage 5) stages, which is an additional decrease of almost 15%. After PVSM, the heart rate increased again in P2 (stage 6) when the participants read a speech text excerpt for the second time.

**Figure 15:**
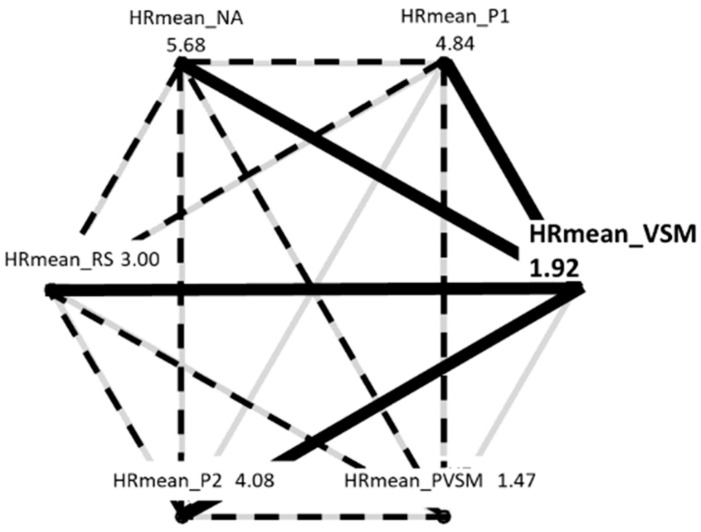
Overview of the paired-comparison t-test results. Thick black lines show significant differences between VSM and other stages (NA, P1, RS, P2); dotted black lines indicate other significant mean HR differences among stages.

Multiple paired comparisons of mean HR levels among stages (with Bonferroni correction for multiple testing and Cohen’s d as a measure of effect size) revealed that each stage differed significantly from the preceding one. That is, the decrease from NA to P1 was significant (t[37] = 4.377, *p* < 0.001, d = 5.31), as was the one from P1 to RS (t[37] = 21.721, *p* < 0.001, d = 3.17). Most importantly, heart rate also decreased significantly from RS to VSM (t[37] = 6.422, *p* < 0.001, d = 5.39), while the VSM stage did not differ from PVSM. Similar to the TBR and BAR results, the HR in the VSM stage differed from both of the P1 (t[37] = −17.862, *p* < 0.001, d = 5.80) and P2 (t[37] = −9.048, *p* < 0.001, d = 8.19) reading stages. Figure 15 is an overview of all significant HR differences among the stages.

#### 3.2.2. Mean HRV

**Figure 16:**
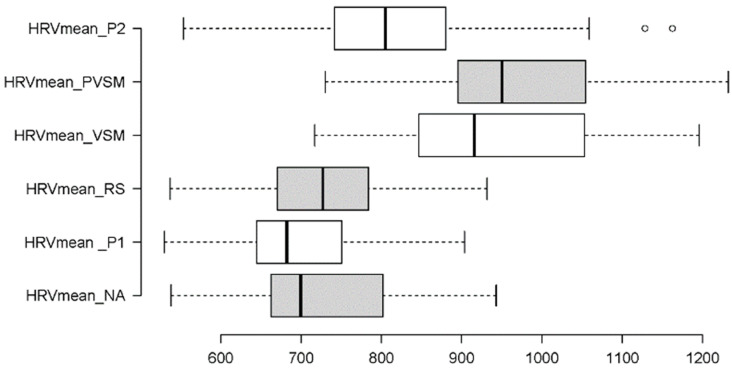
Boxplot summary of mean HRV results for the six stages of this study, shown in chronological order from bottom to top. The horizontal axis shows variability values in milliseconds.

The mean HRV data met all the requirements to be statistically analysed using an RM-ANOVA, based on the six-level fixed factor stage. Like the mean HR, the main effect of stage on the mean HRV was significant (F [5,160] = 2.311, *p* = 0.047, η_p_^2^ = 0.069), albeit to a weaker degree than for the mean HR. Figure 16 shows that the HRV levels increased after the RS (stage 3) by almost 30% and only decreased again after PVSM, during P2 (stage 6).

**Figure 17:**
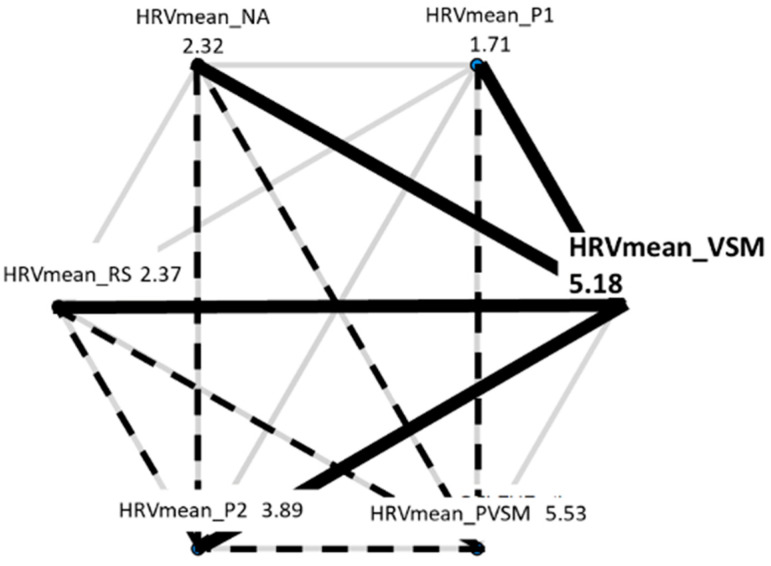
Overview of the paired-comparison t-test results. Thick black lines show significant differences between VSM and other stages (NA, P1, RS, P2); dotted black lines indicate other significant mean HRV differences among stages.

Multiple paired t-tests (with Bonferroni correction for multiple testing and Cohen’s d as measure of effect size) showed that HRV mean differed significantly between RS (stage 3) and VSM (stage 4) (t[37] = −12.404, *p* < 0.001, d = 100.64), as well as between PVSM (stage 5) and P2 (stage 6) (t[37] = 7.120, *p* < 0.001, d = 118.49). In addition, the mean HRV levels during VSM (stage 4) were notably different from the P1 (stage 2) (t[37] = 18.311, *p* < 0.001, d = 77.46) and P2 (stage 6) (t[37] = 6.424, *p* < 0.001, d = 113.51) reading stages. A complete overview of significant paired comparisons is provided in Figure 17.

#### 3.2.3. Sympathovagal Balance: LF/HF Ratio

To measure the activity of the heart and cardiac system, a time and frequency domain analysis was used. The LF/HF ratio is a frequency domain analysis metric from which the sympathovagal balance of the autonomic nervous system can be deduced [97]. It is a ratio between the spectral power of low frequencies (which indicate sympathetic activity) and the spectral power of high frequencies (which indicate parasympathetic activity). A high LF/HF ratio indicates dominance of the sympathetic nervous system [98], and a low LF/HF ratio implies parasympathetic activity and a relaxed response.

**Figure 18:**
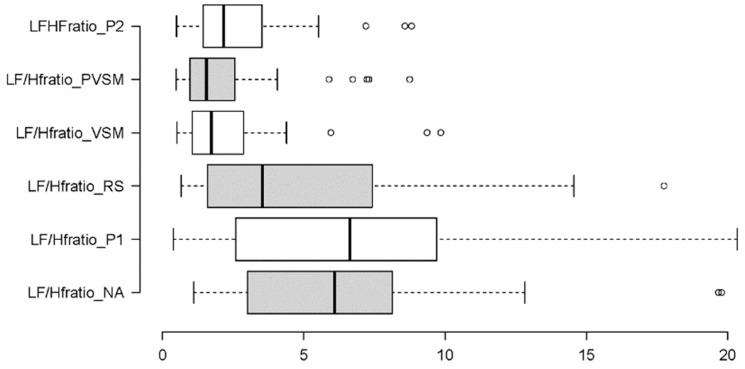
Boxplot summary of LF/HF ratio results for the six stages of this study, shown in chronological order from bottom to top. The horizontal axis shows LF/HF ratios (of two underlying Hz values).

**Figure 19:**
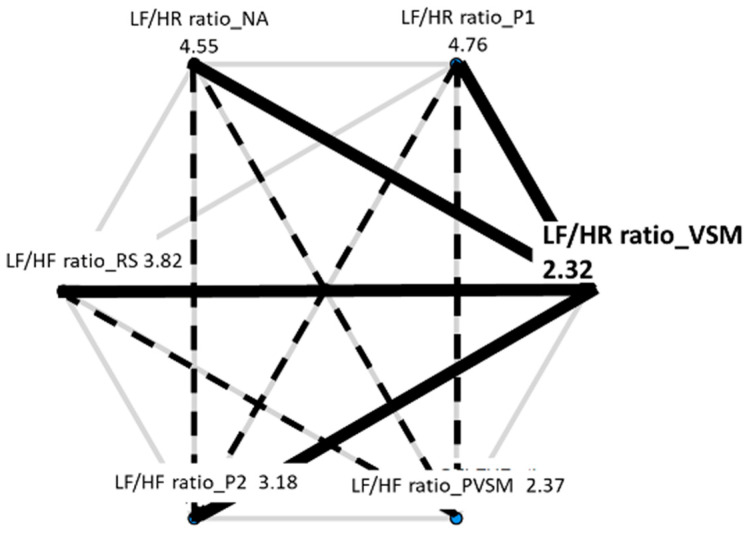
Overview of the paired-comparison Wilcoxon test results. Thick black lines show significant differences between VSM and other stages (NA, P1, RS, P2); dotted black lines indicate other significant LF/HF ratio differences among stages.

To analyse the LF/HF ratio data, the Friedman test was used as a non-parametric pendant of the RM-ANOVA. The main effect of stage was significant (χ^2^[5] = 60.647, *p* < 0.001, W = 0.32). As seen in Figure 18, this main effect was primarily caused by a decrease in the ratio of about 35% from the first reading to the resting stage (P1 to RS). It then strongly decreased further by almost 60% from the resting stage (RS) to the VSM stimulation. According to the additional Wilcoxon tests that were carried out between the levels of stage (with Bonferroni correction for multiple testing and r as an effective size indicator), both decreases were statistically significant (P1-RS: W = 2.207, *p* = 0.027, r = 0.36; RS-VSM: W = 3.495, *p* < 0.001, r = 0.57). Although the increase in the LF/HF ratio after PVSM (stage 5) was small, the difference between VSM (stage 4) and P2 (stage 6) was also significant (W = 2.023, *p* = 0.043, r = 0.33). This shows that on average, the ratio was higher in the second reading stage (P2) than during VSM. Figure 19 provides a summary of all significant differences in LF/HF ratios among the experiment stages.

#### 3.2.4. Sympathetic Activity: SDNN and LF

**Figure 20:**
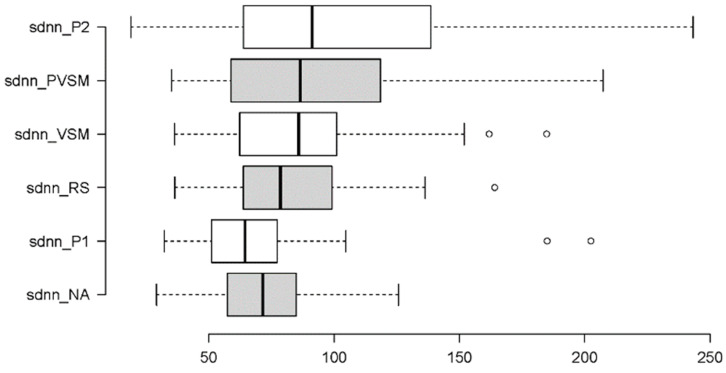
Boxplot summary of SDNN results for the six stages of this study, shown in chronological order from bottom to top. The horizontal axis shows Hz values.

**Figure 21:**
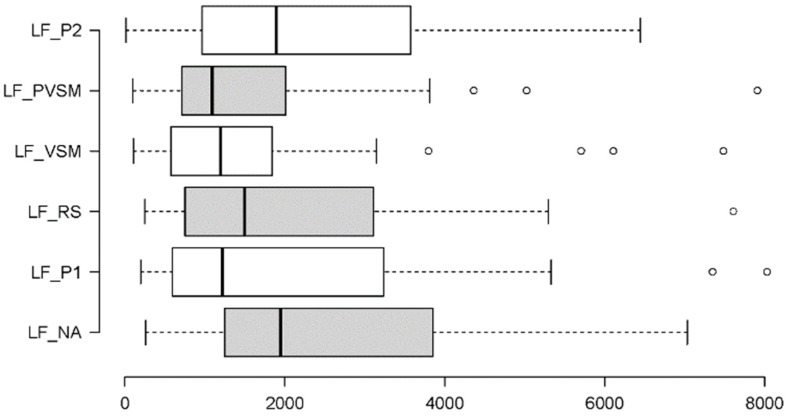
Boxplot summary of LF results for the six stages of this study, shown in chronological order from bottom to top. The horizontal axis shows Hz values.

To understand the sympathetic activity of participants across the study stages, the following measures were implemented: SDNN (a time domain measure of the standard deviation of RR intervals) and LF (a frequency domain measure of the spectral power of low frequencies). Taking into account the distributional characteristics and variability in the data, the first measure, SDNN, was analysed using an RM-ANOVA, while the second measure, LF, was analysed using a Friedman test. In addition to the graphical summaries in Figure 20 and Figure 21, Table 3 summarises the results of the inferential statistics.

Table 3 shows the significant main effects of stage found on both measures of sympathetic activity (SDNN and LF). In combination with Figure 20 and Figure 21, it can be seen that the effects were in opposite directions. For SDNN, there was a stepwise increase in measurements, specifically a tripartition. The first two stages, NA and P1, yielded similarly low SDNN levels. Then, SDNN went up to similar degrees during the next three stages including the RS, VSM and PVSM. The third and last increase occurred for the final P2 stage. Similar to many of the other measures, the key stimulation stage, VSM, differed from both the P1 and P2 reading stages, as well as from the initial stage of normal activity (NA), but not from the surrounding inactive RS and PVSM stages.

In contrast, LF measures did not increase but decreased under VSM stimulation and remained similarly low in the subsequent PVSM stage. Both VSM and PVSM yielded significantly lower LF values than the preceding resting stage (RS). The two reading stages (P1 and P2) caused on average higher participant LF levels than both the VSM stage and the following PVSM stage.

#### 3.2.5. Parasympathetic Activity: RMSSD and HF

**Figure 22:**
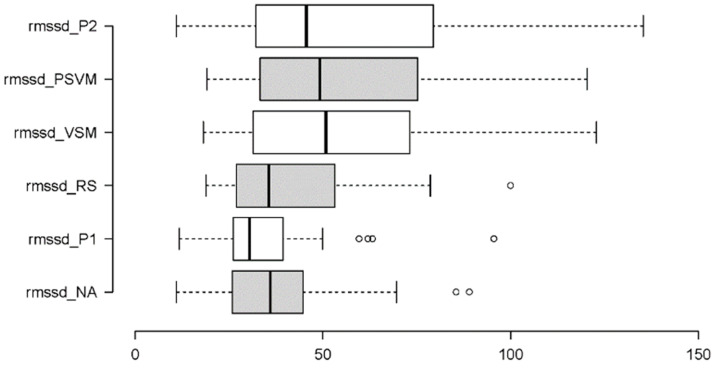
Boxplot summary of RMSSD results for the six stages of this study, shown in chronological order from bottom to top. The horizontal axis shows dB values.

**Figure 23:**
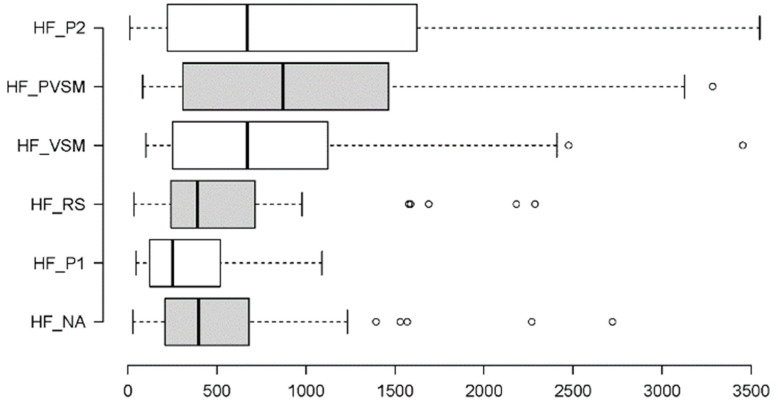
Boxplot summary of HF results for the six stages of this study, shown in chronological order from bottom to top. The horizontal axis shows Hz values.

To determine how the six experimental stages affected participant parasympathetic activity, the following metrics were used: RMSSD (a time domain indicator of beat-to-beat variability between adjacent RR intervals) and HF (a frequency domain metric measuring the spectral power of high frequencies). Two Friedman tests were performed, the results of which are summarised in Table 4. In addition, Figure 22 and Figure 23 provide boxplot summaries of the measurement differences across the six stages.

As shown in Table 4, the independent variable stage had significant main effects on both dependent variables RMSSD and HF. The two effects were strong and similar: RMSSD and HF levels were sustained for VSM (stage 4) and throughout the last two stages (PVSM and P2). This differed from the preceding NA (stage 1) and P1 (stage 2) stages. Unlike most of the other measures, there was no notable difference among the VSM measurements of these metrics compared to the PVSM and P2 stages.

Figure 22 shows that RMSSD increased across the six stages. More specifically, it increased from the resting stage (RS) to the VSM stimulation stage. All three stages before VSM were statistically indistinguishable in terms of RMSSD. The same was true for the VSM stage and the two following stages.

For the HF measure, Figure 23 illustrates a significant change from the resting stage (RS) to the key stimulation stage (VSM) and the two subsequent stages (PVSM and P2). There was also a bipartition of the HF measurements across the six stages of the experiment. The dividing line is between the first three phases (NA, P1 and RS) and the last three phases of the study (VSM, PVSM and P2), where an increase in HR values occurred during the VSM phase and during the following two phases.

However, unlike RMSSD measurements, the participants’ HF levels were already relatively high in the initial normal activity stage (NA), which is why no significant increase was found in HF levels between the NA and VSM stages.

### 3.3. Psychological Stress

To understand if VSM benefits individuals with higher perceived psychological stress, the following quantitative dependent variables were considered: TBR, BAR, FAA, mean HR, mean HRV, LF/HF ratio, SDNN, LF, RMSSD, and HF. For each of these metrics, participant data were calculated individually as the difference between the measured value during VSM (stage 4) and the value measured during the NA condition (stage 1). Thus, if VSM had a large effect on perceived psychological stress, then the difference in values between VSM and NA would be large.

Total PSS-10 scores from all 38 participants were individually correlated with their respective VSM-NA difference values. Because of the distributional characteristics of the data, non-parametric Spearman’s Rho correlations were used. Table 5 summarises the results of these correlation tests. The first row shows the measures (dependent variables) for which the ten VSM-NA difference values were determined. The second row contains the correlation coefficients, which indicate how the difference values are related to the PSS-10 scores of the 38 participants. The third row shows the *p*-values associated with the correlation coefficients. Significant correlations are marked by asterisks.

There were two significant, negative correlations, one concerning SDNN and the other one concerning LF. Note that sympathetic activity is reflected in reduced SDNN and LF values. Thus, negative correlations of SDNN and LF with PSS-10 scores mean that participants with higher PSS-10 scores produced smaller VSM-NA difference values for SDNN and LF. Smaller VSM-NA difference values mean lower VSM values. The correlations can hence be summarised as follows: relative to the initial NA condition, the higher the perceived psychological stress level of a participant before VSM stimulation, the more the VSM stimulation reduced the participant’s sympathetic nervous system activity. It is important to note that although sympathetic nervous system activity decreased, parasympathetic activity did not increase.

In addition to the correlations, a series of between-subject comparisons were conducted using Mann–Whitney U-tests. Using the average score of all participants as a dividing threshold, each participant was assigned to either a Low-Stress or High-Stress group. The Low-Stress group included participants with a psychological stress score of less than or equal to 24 (on a scale of 0–50, N = 22). The High-Stress group included those with a psychological stress score of 25 and higher (N = 16). The results of the U-tests supported the correlations found. The participants with higher total stress scores (≥25) had significantly smaller VSM-NA difference values than those with lower total stress scores (≤24). This was true for both SDNN (U[22,16] = 249, z = 2.158, *p* = 0.031) and LF (U[22,16] = 246, z = 2.070, *p* = 0.038).

**Figure 24:**
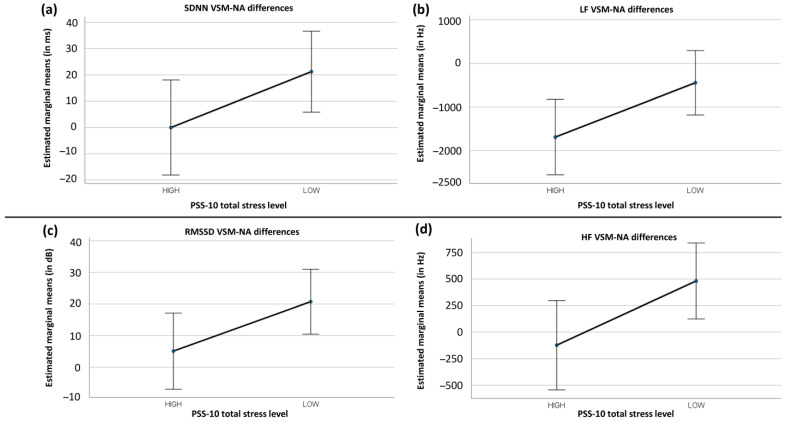
Significant differences in physiological stress measures between participants with low levels (total scores ≤ 24) and high levels (total scores ≥ 25) of perceived psychological stress, estimated based on the PSS-10 questionnaire. SDNN ((**a**), top left), LF ((**b**), top right), RMSSD ((**c**), bottom left), and HF ((**d**), bottom right).

The top two panels (a) and (b) in Figure 24 illustrate how the two psychological stress groups differ in SDNN and LF when their NA measurements are subtracted from their VSM measurements. Although the results look similar initially, the following aspect must be noted: in the case of LF (Figure 24b), the VSM-NA difference values dropped from an average of about 0 (Low-Stress group) to a clearly negative value range (High-Stress group). In contrast, the difference values for SDNN (Figure 24a) decreased from a positive value (Low-Stress group) to a value of almost 0 (High-Stress group). This means that, although both SDNN and LF were significantly reduced by VSM stimulation, only the LF values for the High-Stress group fell below 0 and, thus, below the level of the initial NA stage. This indicates a positive effect of the VSM treatment in terms of reduced activity of the sympathetic nervous system for the High-Stress group.

The two lower panels (c) and (d) in Figure 24 show two further stress group differences for the RMSSD and HF measures from which no significant correlations were obtained. The RMSSD difference values again relied on a U-test result (U[22,16] = 249, z = 2.158, *p* = 0.031) and the HF difference values could be analysed in a univariate ANOVA (F[1,36] = 4.958, *p* = 0.032, η_p_^2^ = 0.121). Results of RMSSD and HF fell from a clearly positive range of values for the Low-Stress group to a range of values close to 0 for the High-Stress group. This means that, compared to their initial NA values, VSM stimulation had essentially no effect on the High-Stress group, in terms of increasing their RMSSD and HR values. In contrast, the Low-Stress group did benefit from VSM exposure in the form of higher RMSSD and HR values (both of which are markers of parasympathetic activity).

## 4. Discussion

The aim of this study was to investigate the nature and effectiveness of a vibroacoustic technology called Vibroacoustic Sound Massage (VSM). This research tested a vibrotactile bed-like device [43] on which participants laid in the supine position for a 45 min stimulation. They were stimulated with both tactile vibration and acoustically through ambient soundscapes tailored to and time-aligned with these vibrations.

Three research questions were tested. Research Question One (RQ1) addressed the effects of cognitive stress, i.e., how does VSM affect mental processing? The following hypothesis (H1) was associated with this question: VSM has positive effects on user concentration, relaxation, and well-being. During VSM stimulation, it was assumed that participants would feel more concentrated while being in a more relaxed state, and would feel improved overall well-being. In terms of EEG measures, this would mean a lower TBR level, a lower BAR level, and a higher FAA level.

Research Question Two (RQ2) assessed physiological stress, i.e., how does VSM affect the autonomic nervous system, vagal tone, and heart rate? The corresponding hypothesis (H2) was as follows: VSM lowers physiological stress, increasing parasympathetic and decreasing sympathetic nervous system activity. With respect to the applied measures, this would manifest as an increase in mean HRV and a decrease in mean HR. Increased RMSSD and HF levels were also expected, as well as reduced SDNN and LF levels.

Research Question Three (RQ3) dealt with psychological stress, i.e., are participants with higher PSS-10 scores more impacted by VSM? The following hypothesis (H3) was put forward: VSM benefits individuals with higher perceived psychological stress. It was assumed that participants with higher PSS-10 scores (the High-Stress group) would show more pronounced VSM effects in terms of H1 and H2.

The experimental sessions consisted of six stages. This enabled a thorough investigation of the three research questions and hypotheses from different angles and allowed us to collect data beyond the three research questions and hypotheses addressed in the present paper; see Fooks & Niebuhr (2024) [85]. Stage 1 (NA) served as a control reference or baseline condition. As this research was conducted in Copenhagen, Denmark, it was likely that the participants biked to the study location. NA enabled participant heart rates to return to a more normative restful state. However, NA required participants to sit and listen, which is not the most natural “normal activity”. Stage 2 (P1) matched the naturalness criterion more appropriately as the participants sat and read a text aloud. A study by Emanuel et al. (2008) [99], which also used students as the main participant group, showed that listening took up about the same amount of time in students’ everyday lives as reading and speaking. Therefore, P1 was regarded as a second and arguably more realistic baseline condition for a student participant sample. Stage 3 (RS) was a resting control state for participant HR before VSM stimulation in stage 4. Additionally, if and how the RS stage differed from the VSM stage and the NA and P1 control stages, respectively, indicated whether VSM effects could be ascribed to the VSM stimulation itself or whether the results were simply the effect of several minutes of rest in a comfortable supine position with closed eyes. Stage 5 (PVSM) supported the estimation of the sustainability of VSM stimulation effects: the differences between VSM and PVSM determined the degree to which VSM effects continued beyond the end of the stimulation itself. During stage 6 (P2), the participants read aloud the same text as in P1. Neither the P1 nor P2 conditions were analysed in the present study, although a discussion of these results can be found in Fooks & Niebuhr (2024) [85].

### 4.1. Discussion of Cognitive Stress Effects

The participants’ TBR levels (theta/beta ratio, a metric of concentration and focus) dropped significantly in the VSM stage, both in relation to the second P1 reference condition and the preceding RS resting condition. In the subsequent PVSM stage, the TBR remained low before increasing significantly again in the P2 stage. The following conclusion can be drawn from this result: VSM had a concentration-enhancing effect. This effect was genuine as it differed in the same way from the RS stage as from the reference condition P1. The difference compared with the preceding RS stage indicated that lying on the device in a comfortable, supine position with closed eyes was not solely responsible for the VSM effect on TBR. The effect was sustained because the TBR level remained consistently low during the PVSM stage, while subsequent activity after this (during P2) reversed the effect. The reversal was complete because the TBR levels in P2 did not differ from those in the initial NA and P1 reference conditions. These TBR results indicate that mind-wondering diminished and concentration or focus increased. This finding is supported by research showing that vibroacoustic stimulation can evoke a cognitive flow state [100]. The results of the present study do not imply that the participants were concentrated on daily tasks or practicalities, but rather that their focus was toward the experience of VSM itself. The verbal first-person accounts are consistent with this, with the participants reporting feeling creative and an ability to “zone” in and out of thoughts (see Table 2). On many occasions, a psychedelic trip-like state was described, alongside “seeing” changes in light and colour in sync with the music and vibration (see *Section 3.1.4 Qualitative data: First-person Verbal Accounts*). Cognitively, VSM seems comparable to a 3D planetarium experience as the participants were perceptually removed from everyday routine with their locus of attention towards an immersive experience. As their eyes were closed throughout, the only perceptual avenues the participants were able to access were tactile and audible. The TBR results and verbal accounts together indicate that the focal point of concentration was an embodied, immersive perceptual VSM experience. More research is required to better understand what attributes of VSM (auditory, tactile, or both) caused this outcome.

The participants’ BAR levels (beta/alpha ratio, a metric of arousal) dropped significantly in the RS stage, remaining at that level during the VSM stage and in the subsequent PVSM stage. All other surrounding activity stages (NA, P1 and P2) showed higher BAR levels. This leads to the following conclusion: the expected effect of VSM on BAR was found, i.e., the participants became less aroused and more relaxed. Crucially, however, there is no evidence of a separate relaxation effect of VSM stimulation. That is, a diminished BAR level was sustained across the RS, VSM, and PVSM stages, which implies that the combination of closed eyes and a quiet, lying position caused lower BAR levels. Notably, there is no evidence in the data that VSM stimulation negatively interfered with the relaxation levels obtained by participants initially during the RS stage. This reduction in the arousal and relaxation effects was completely reversed in the P2 stage, as the BAR level immediately increased to the level of the NA and P1 reference conditions. With very little variation among participants, these BAR results indicate that all participants remained relaxed to similar degrees during the RS, VSM, and PVSM stages.

There were no significant effects of the FAA level (frontal alpha asymmetry, a metric of well-being). This result indicates that neither VSM stimulation nor the surrounding rest phases could demonstrably increase the well-being of the participants. This result may be due to inter-individual participant variables, and a larger participant sample is required in future studies for result generalisability. However, the average variability in FAA was reduced by VSM. That is, the participants with low levels of well-being prior to VSM exposure ended this study feeling a lot better, while the participants starting this study with high levels of well-being sustained their levels. A larger participant sample is required to gain statistically significant FAA results.

In summary, hypothesis 1 (H1) can be partially accepted. There was clear empirical evidence that VSM stimulation can positively influence cognitive stress and induce higher levels of concentration. VSM stimulation did not have a negative effect on relaxation, but the type and strength of the relaxation effect could not be distinguished from other stages of rest when lying down with closed eyes. Well-being was not significantly increased through VSM stimulation, although variability in this metric was homogeneously reduced.

### 4.2. Discussion of Physiological Stress Effects

The measurement parameters for physiological stress were multifaceted. As the essential effects on all were similar, in this section, they are discussed together. Most importantly, stimulation effects were obtained in the direction expected by hypothesis 2 (H2) except for SDNN. Additionally, the results indicated that many effects of VSM also stood out from the previous RS stage. This suggests that the VSM stimulation had genuine, beneficial effects on physiological stress that went beyond what could be achieved by a comfortable supine position and closed eyes alone. The participants’ heart rates decreased further in the VSM stage when compared with the RS stage, and a shift towards more parasympathetic than sympathetic autonomic nervous system activity was more pronounced in the VSM stage than in the previous RS stage. Expectedly, though less categorically, the opposite was true for the HR. The HR was higher in the NA and P1 stages, decreased during the RS, and considerably dropped during VSM and PVSM. Increasing only marginally for P2, all the participants were more physiologically relaxed at the end of this study than at the beginning. The mean HR during VSM and PVSM was comparable to that of a healthy sleeping adult (around 60 BPM), demonstrating that VSM induces a restful physiological state.

Hypothesis 2 predicted an increase in HRV RMSSD and HF values (indicative of parasympathetic activity), which was supported by the results. Throughout NA, P1, and RS, lower levels of both metrics were sustained before increasing during VSM and remaining high throughout the PVSM and P2 stages. This shows that VSM onset categorically increases RMSSD and HF. These results effectively illustrate that VSM increases parasympathetic activity. The metrics of sympathetic activity were less significantly affected. The results showed that LF was marginally decreased, while SDNN in fact increased. Compatible with hypothesis 2, LF gradually decreased in NA, P1, and RS and remained low throughout VSM and PVSM before increasing again at P2. The rise during P2 could be due to the condition demanding concentrated reading. It is likely that the stark contrast between a relaxed state and a focused reading task spiked sympathetic activity. Similarly, participants read aloud with a researcher present in the room, which had the potential to heighten physiological and cognitive alertness. Like LF, SDNN also decreased in NA, P1, and the RS before unexpectedly marginally increasing in VSM, PVSM, and P2.

With regard to the sustainability of these beneficial effects of VSM stimulation, the results are as clear as they are homogeneous across all parameters. None of the physiological stress parameters showed a reversal of the RS-to-VSM changes in the PVSM stage. The positive physiological effects of VSM stimulation remained qualitatively and quantitatively intact even in the subsequent rest phase. Even when the participants became active again during the P2 stage, most parameter levels did not bounce back to their original levels in the NA and/or P1 reference conditions. The parameters that partially retained the positive physiological effects of VSM stimulation in the P2 stage were mean HR, mean HRV, the LF/HF ratio, LF, RMSSD, and HF.

In summary, hypothesis 2 (H2) can be fully accepted. The only exception was the SDNN parameter, which did not evolve in the same direction as the LF parameter. It was not possible to determine why SDNN was an exception or why it differed even from its neighbouring measure, i.e., LF. However, because of the complexity of the physiological stress measurements and the fact that the SDNN results were not only contradictory to LF and all other parameters but also weaker overall, there is no need to over-interpret the SDNN results in the sense of undermining H2. This result may be due to the robustness of the metric with regard to sensitivity [101]. Future studies could consider only the HF and LF parameters to avoid similar result patterns; however, as these parameters have been shown to decline in human subjects with age [102], appropriate metrics need to be implemented with respect to study design.

### 4.3. Discussion of Psychological Stress Effects

The data did not provide a consistent answer to whether people with high levels of psychological stress benefit more from VSM stimulation than others. There were two significant (negative) correlations in the participant PSS-10 scores that were in line with this hypothesis (H3). First, the VSM-to-NA differences of both SDNN and LF (metrics of sympathetic activity) indicate that the higher a participant’s self-determined psychological stress level, the more VSM stimulation was able to reduce that participant’s SDNN and LF levels. However, the correlations were weak, and the levels of explained variance were low. Additionally, only the VSM-to-NA differences in LF fell on average below 0. This means that even though SDNN was negatively correlated with PSS-10 scores, SDNN levels for the participants with high PSS-10 scores were still higher in the VSM stage than in the NA stage. When participant PSS-10 scores were high, VSM stimulation reduced only the LF metric below those of the NA stage. This result indicates that VSM had a less profound effect on the parasympathetic activity of those in the High-Stress group. Thus, the High-Stress participants responded less well to VSM.

Additional paired comparisons between the High-Stress and Low-Stress groups revealed two further differences in RMSSD and HF (parasympathetic activity metrics). These differences suggest that participants with low PSS-10 scores benefitted more from VSM stimulation, experiencing more parasympathetic activity than participants with high PSS-10 scores. These results contradict hypothesis 3 (H3), which predicted that VSM would benefit individuals with higher perceived psychological stress. Overall, the results are inconclusive with regard to hypothesis H3 and are limited solely to measures of physiological stress. There were no effects at all for the parameters of cognitive stress; thus, the hypothesis must be rejected rather than accepted.

Future studies could consider using the PSS-10 as a subjective measure of psychological stress, as well as an additional objective measure, to provide a more rounded assessment of participant baseline stress levels. It is likely that VSM stimulation has different effects on different people, relative to their personal background and living conditions. For example, VSM may have a stronger and/or more positive effect on parasympathetic activity for people with lower PSS-10 scores, whereas, for people with higher PSS-10 scores, it may have a stronger and/or more positive effect on sympathetic activity. That is, people who perceive their life predominantly as unpredictable, uncontrollable, and overloading, could benefit from VSM by a mitigated “flight-and-flight’ response rather than an enhanced “rest-and-digest” nervous system response. An additional objective stress metric would support the objective effects of the intervention on individuals, irrespective of their own perceived experiences of stress.

The authors are currently conducting a follow-up study [103] that assesses the effects of inter-individual lifestyle differences on VSM stimulation. The research implements the International Physical Activity Questionnaire (IPAQ) [104] to understand how and if physical activity levels determine VSM efficacy (with respect to the regulatory effect of exercise on nervous system activity and HRV metrics). Additionally, the study aims to understand if participants who engage in higher levels of mindfulness are more or less affected by VSM. To this end, the study integrates a self-report measure of perceived mindfulness, the Freiburg Mindfulness Inventory (FMI-13) [105].

### 4.4. Relevance and Implications

In addition to our first study evaluating the effects of VSM on the biosignal of voice in a read-speech task [85], this research provides further evidence in favour of the effectiveness of VSM. While changes in speech are behaviour-based, this study directly quantifies the effects of VSM using EEG and ECG, i.e., biosignals that do not demand behavioural manifestation. The present data show, in greater detail than speech analysis data, that VSM can reduce user cognitive stress and aid concentration. Additionally, the main positive effect of VSM concerns physiological stress, which is reduced by modality. Contrary to the effects on cognitive stress, the reduction in physiological stress from VSM lasts well beyond the stimulation period. Future research is required to determine precisely how long this positive physiological stress effect lasts. It should be emphasised that the damping effect that VSM has on physiological stress is of great social benefit, as physiological stress is a driving force for depression [106,107,108] and a number of cardiovascular diseases from hypertension to heart attacks to insomnia and obesity [109,110,111,112,113]. These results provide further evidence in support of the utility of vibroacoustic VSM technology.

In the context of physiological stress management, the results of the present study show that VSM reduces heart rate and increases heart rate variability which together indicate a heightened “rest-and-digest” nervous system response and strengthened vagal tone, which supports well-being [114,115]. This effect of VSM is shared with other stress-reducing interventions including yoga and mindfulness meditation, both of which are well-established stress management tools [6,116]. All three interventions increase HRV, which is illustrative of less physiological stress and a relaxation response [3,117,118]. Further research is required to better understand what VSM elements most efficaciously increase HRV. Specific questions include the following: to what degree do sonic factors (which sounds and/or frequencies) have an effect as compared to vibrational force? Is there a marked difference in the physiological effects of these factors, and if so, to what extent? Future studies could consider comparing VSM with a sound bath. Sound baths are meditative well-being interventions in which participants lay in the supine position and listen to live instrumentation, often of Tibetan origin, similar to that used in the VSM soundscape [119]. During sound baths, physical contact between instruments and recipients is rare, though they share the stress-reducing physiological effects of VSM and mindfulness meditation [120]. A comparison study between VSM and a sound bath using the same audio elements for both interventions could test the extent to which kinetic tactile vibration affects HRV and HR during VSM exposure. As evidenced, the physiological stress effects of VSM are comparable to existing stress-reducing interventions, but more exploration is necessary.

With respect to neurological stress management in the present study, the TBR results indicate that mind-wandering reduced and concentration increased during VSM, which aligns with the effects of mindfulness meditation [121]. This finding highlights how VSM shares beneficial attributes with a well-being tool widely used for its stress-reducing qualities. The BAR results show that relaxation increased from VSM, which is in agreement with the effects of two well-adopted stress-reducing interventions, i.e., yoga and mindfulness meditation [122,123]. The TBR and BAR results suggest that VSM may affect the same underlying neural mechanisms as these two interventions. So, although there were no significant well-being effects with respect to the FAA results, as VSM shares other outcomes with two stress-relieving and well-being-improving interventions, additional VSM studies are required. A larger participant sample will increase our understanding of if and how VSM can improve well-being. As evidenced, the cognitive and physiological parameters used in this study are adept stress measures that are well-positioned for further research in this field in addition to other metrics. Upon deeper exploration, the physiological and cognitive effects of VSM are shared with two established stress-reducing practices, and further exploration would improve our understanding of how VSM can affect stress and the extent to which it has utility as a stress management tool.

The inventors of VSM technology claim that it is “immediately relaxing [to] the mind and body, eliciting feel-good emotions, an increased state of well-being and improved mind-body connection” [124]. They further state that their technology is “legitimising the power of sound vibration therapy as an effective and non-invasive Vagus Nerve Stimulation (VNS), activating the parasympathetic nervous system, the ‘rest and digest’ state” [124]. However, the results of this study are not entirely consistent with these claims. On the one hand, our data clearly demonstrate an effective and non-invasive Vagus Nerve Stimulation (VNS) in addition to a resulting shift towards greater parasympathetic nervous system activity. But the data also indicate that inter-individual differences determine the degree of its effectiveness, as evidenced in the PSS-10 scores; however, this metric as a subjective stress measure may not accurately reflect true stress levels. Future research could consider using this metric with additional robust objective stress measures to validate the results. Additionally, these findings, like those of Fooks & Niebuhr’s (2024) speech-signal analysis [85], contain no objective (EEG) evidence of stimulated feel-good emotions or a heightened state of well-being. Of course, it is possible that the EEG measurements were not sensitive enough to support these claims with data, or it may be that the experimental study conditions affected the VSM outcomes. With regard to the latter, all research conducted into vibroacoustic technology is limited outside of a clinical setting. This could be remedied by more non-clinical trials

As with all prospective treatments, discussions must be had in the healthcare sector among policymakers, medical practitioners, and patients as respective end-users, though there is space for further research in this field. Potential implications could see the conception of specialised spaces in clinics and/or hospitals set up for VSM treatment. Additional measures, in situ testing, extensive debriefing questionnaires, and/or hospital-grade EEG device data would also help resolve empirical uncertainties.

Finally, a subsection on implications must also address the heterogeneous variances. Statistically speaking, they are not a validity problem, especially with larger samples and for distributions that are not excessively skewed [125]. Both are true here. In terms of interpretation, there are several causes of these heterogeneous variances. What is important here is where these variances occurred, namely, between stages 1 and 6. Therefore, one reason lies inherently in the experiment design. It is known that different levels of task demands or stimulation complexity lead to different variances [126]. The variance within a sample usually increases with increased task demands or stimulation complexity. Our RMSSD and HF data could be an example of this because, from the VSM stage onwards, the variance increased considerably. But not all the results of all the measures can be explained in this way. In some cases, the variance increased successively over the six stages, for SDNN and, to a certain extent, TBR. Fatigue, stress or, more generally, changes in the level of motivation could be the cause of such successively increasing variances. The opposite is true for successively decreasing variances, as in the case of our FAA and LF/HF ratio variables. Such motivation-related fluctuations are not a problem here since the participants did not have to complete a specific assessment, reaction time, or behavioural task.

Whatever the underlying cause, heterogeneous variances are always the result of enhanced or reduced inter-individual differences within a participant sample; therefore, they are a reason to investigate the inter-individual effects of VSM and the adjacent resting states more closely. An ongoing follow-up study is currently underway (see Section 4.5) with shorter experiment and VSM stimulation durations in order to mitigate any stress or motivational fluctuations while, at the same time, increasing the number of participants.

### 4.5. Limitations and Outlook

The present study was limited in a select few ways. Firstly, the sample size of 38 participants was relatively small and focused mainly on a select group of educated participants (primarily young, educated university students). Including and balancing additional participant variables such as ethnicity, socioeconomic status, and gender would provide a more diverse sample and further comprehensive insights. However, the focus of the present study was not on participant-specific VSM effects (these will be dealt with in greater detail in a follow-up study), but on participants’ lifestyles and how they relate to stress and well-being in connection with VSM stimulation. That is, the fundamental question here was the following: is VSM effective at all and in what way? Moreover, as outlined in Section 2.2.5, there is no empirical reason to assume that gender would have an effect on the present data, and, most importantly, there is hardly any evidence for qualitative gender differences in EEG and heart rate measures. In addition, our within-subject design ensured that the differences obtained could not be caused by imbalances between the compared subsamples. Yet, the present sample is evidently not representative of the general public; thus, the results are not generalisable outside this demographic. Therefore, the results are not representative of the future target customers for VSM. Thus, more research with a larger and more diverse participant pool needs to be conducted to gain broader, more nuanced insights into the modality and its effects. This research is currently underway as previously described.

Secondly, the present study also collected additional lifestyle meta-data (into exercise routines, sleeping, and smoking habits, mindfulness practises like meditation, and perceived musicality insights), none of which were analysed here. An analysis of these meta-data collated with the results discussed in this paper will aid in further comprehending VSM effects, which will be available in a future paper.

Thirdly, the 45 min VSM stimulation time in the present study is not a practical duration for many, neither for individual users nor for companies that would like to offer VSM during work breaks. The authors are currently conducting a study using the same VSM technology with a shorter stimulation time of 20 min to assess if the effects are still present. Future research could be conducted to determine at what point VSM effects are optimal by measuring both their onset and duration. This would provide insight into the minimum stimulation time required for VSM, which would be beneficial for end users and for the future development of vibroacoustic products.

Fourthly, very limited speculative conclusions can be drawn from these data with respect to long-term effects. Future longitudinal studies need to be conducted to better understand the prolonged effects of vibroacoustic interventions. These studies could measure treatment regularity, which would show if there are cumulative or diminishing returns from repeated exposure, as well as any potential habituation effects of VSM.

Fifth, additional studies specifically comparing vibroacoustic technology with Vagus Nerve Stimulation (VNS) need to be conducted to define it either as an adjunct treatment or replacement with similarities and differences explicitly defined between the two.

Sixth, this study lacks a control group, which poses a challenge when isolating the specific effects of VSM from other potential variables. Future research could implement control and/or placebo conditions to rule out the placebo effect as a contributor to the observed results. Including a sham intervention group would help address this limitation.

Seventh, an exploration of the effects of different types of vibroacoustic stimuli, such as varying frequencies, soundscapes, and levels of vibration, could help determine the most effective parameters of the intervention.

Lastly, the resting and stimulation stages, i.e., RS, VSM and PVSM, showed more significant differences overall compared with the P1 stage than with the NA stage. This indicates that P1 is a more appropriate reference control condition than NA, which is likely due to inter-individual participant variables. Future studies should consider using even more accurate natural control baseline measures as reference markers for all participants that sustain study ecological validity.

## 5. Conclusions

Overall, the results of the present study show that VSM outcomes are not affected by age (within the participant sample age range between 20 and 35 years old). Hypothesis 1 was partially validated, as VSM affected concentration (TBR), arousal (BAR), and homogeneously reduced variability in participant well-being (FAA). Concentration (TBR) increased during VSM, with the results indicating a reduction in mind-wondering and improved focus. Significant reductions in alertness and arousal showed that VSM increased relaxation (BAR), in addition to the two rest conditions before and after the VSM study stage. Though significant results were not found with respect to well-being improvement (FAA), VSM homogeneously reduced the variation in well-being for all participants. With respect to hypothesis 2, VSM increased parasympathetic activity (indicated by increased RMSSD and HF) and marginally decreased sympathetic activity (lowered LF and increased SDNN). Hypothesis 3 was disproven as the PSS-10 scores indicated that VSM was no more effective for individuals experiencing high stress compared with the Low-Stress group. Unexpectedly, VSM increased parasympathetic activity for the Low-Stress group. Collectively, these results indicated that VSM effectively reduced physiological and cognitive stress, though it was not as effective for the High-Stress group.

Our recommendations for future research are broad, including a larger participant sample size and age scope; alterations in stimulation length to assess whether shorter exposure has similar effects; longevity testing to measure for sustained longitudinal outcomes; inclusion of meta-data for comparison with EEG and ECG metrics; implementation of additional qualitative analysis tools such as questionnaires and first-person verbal accounts to deepen subjective insights; inclusion of musicality metrics like the Ollen Musical Sophistication Index (OMSI) and the Goldsmiths Musical Sophistication Index (Gold-MSI) to determine how individual ability and musical preference may affect VSM outcomes; and measurement of different sounds, frequencies, and rhythms to qualify the most effective outcomes of the intervention. Future implications for vibroacoustic development may include the introduction of custom-made vibroacoustic soundscapes tailored to individual preferences and needs.

Contemporary societal pressures and psychologically detrimental platforms (such as new technologies and social media) together support rising discontent and global mental health diagnoses. Thus, new tools to reduce stress and improve sympathovagal balance that are cost-effective and non-invasive are on a highly sought-after trajectory. Further development of vibroacoustic technology could leverage it as a viable tool to support shifting societal needs; however, more research required to quantify its multifaceted effects.

## Figures and Tables

**Table 1:** Study design protocol.

Stage	Duration (Minutes)	Eyes	Posture	Activity	Purpose
Stage 1 Normal activity **(NA)**	5	Open	Sat, chair.	Study introduction.	Neutralise elevated heart rate.
Stage 2 Prosody 1 **(P1)**	5	Open	Sat, VSM module.	Reading speech passage.	Speech prosody analysis. (not discussed).
Stage 3 Rest State **(RS)**	2	Closed	Supine position, VSM module.	No activity required.	Control for basal heart rate, physiological stress, and cognitive stress.
Stage 4 VSM stimulation **(VSM)**	45	Closed	Supine position, VSM module.	Vibroacoustic Sound Massage and vibrotactile soundscape.	Independent variable.
Stage 5 Post-VSM **(PVSM)**	5	Closed	Supine position, VSM module.	No activity required.	Durational measure for VSM effects.
Stage 6 Prosody 2 **(P2)**	5	Open	Sat, VSM module.	Reading speech passage.	Speech prosody analysis. (not discussed).

**Table 2:** EEG relevancy of first-person verbal accounts.

Theta/Beta Ratio (TBR)—Concentration and Focus	Beta/Alpha Ratio (BAR)—Arousal and Relaxation	Frontal Alpha Asymmetry (FAA)—Well-Being
“I craved rhythm, interesting how rhythm doesn’t come through aurally, but physically through vibrations, body craves it, then gets it”	“Not awake but not sleeping, in-between”	“Cathartic”
“Could really match my breath work”	“Hyperactive in my head, rested in my body”	“Really relaxing”
“Not thinking about anything, zone into how much my bodies feeling”	“Disconnected from my body”	“Extremely empowering”
“Zoning into sounds themselves”	”Felt like flying translated into your body”	“Very pleasant”
“Sometimes my thoughts were somewhere else, going in and out of zones”	“After the vibrations it feels numb in a good way, you can’t feel the borders of your body”	“Felt familiar, reminded me of home”
“Mind wondering but still aware of a lot of things”	“It felt liberating”	“I feel super calm and open”
“Pulled down again by thought”	“Complete serenity”	“Clear mind-body connection”
“Distracted me from (ADHD) medication, thinking ‘what is happening with my body?’”	“Body felt super relaxed and calm”	“100% enjoyable”
“There were phases when I was really relaxed, couldn’t think about anything, then all of a sudden really aware”	“Feeling of wanting to transcend, feeling my whole—a unity between my mind and body”	“My tension and stress feels released”
“Once in a while came back to focusing on what’s going on, the music, vibrations”	“Takes away the racing mind”	“Sounds were very calming, especially the birds”
“Totally immersive”	“Feel like you’re floating”	“Nice waves sounds, all ambient relaxing sounds”
“Felt super creative”	“No idea what kind of state I was in”	“Felt like a weighted blanket—safe and comfortable”
“Saw shapes, forms and colours that followed the vibrations in music”	“Vibrations going through the body”	“The vibration resonated with my body deeply”
“I saw colours that made sense with the sounds”	“The stronger the vibration the more relaxed I was, I couldn’t think”	“Feel physically something in my body taken out of me—I feel very light”
“Don’t know where my head went, felt I was tripping”	“The more vibration, the less aware I was”	“A yummy space—feels like a good place—like you’re being mothered somehow”
“Felt like a psychedelic trip”	“Lost track of time”	“Felt like being in my mother’s womb—very safe”
“I felt medicated or drugged in some way”	“Seeing the sound waves going through my body in dancing waves”	“Delightful—reminded me of being a child”
	“Blurred lines between otherness and myself”	
	“Felt I was letting go of thoughts, allowing the experience and sound to come over me lead to deep relaxation”	
	“Stimulated—really relaxing but intense at the same time”	

**Table 3:** Test statistics (left) and multiple-comparisons tests (right) for the two dependent variables SDNN (top) and LF (bottom).

Main Effect of	Test Statistics	*p*	Effect Size	Sig. Multiple Comp. Tests	Test Statistics	*p*	Effect Size
**SDNN**	F [5,160] = 7.267	<0.001	η_p_^2^ = 0.164	VSM vs. NA	t[37] = 2.062	0.046	d = 36.78
				VSM vs. P1	t[37] = 2.075	0.046	d = 42.04
				VSM vs. P2	t[37] = −2.266	0.030	d = 51.84
				NA vs. PVSM	t[37] = 3.746	0.001	d = 8.26
				NA vs. P2	t[37] = 3.463	0.001	d = 7.81
				P1 vs. RS	t[37] = 1.987	0.050	d = 7.50
				P1 vs. PVSM	t[37] = 4.459	<0.001	d = 7.09
				P1 vs. P2	t[37] = 3.855	<0.001	d = 7.18
				RS vs. P2	t[37] = 2.777	0.009	d = 4.61

**LF**	χ^2^[5] = 21.038	<0.001	W = 0.11	VSM vs. NA	W = 4.108	<0.001	r = 0.67
				VSM vs. P1	W = 2.269	0.023	r = 0.39
				VSM vs. RS	W = 2.453	0.014	r = 0.40
				VSM vs. P2	W = −3.004	0.003	r = 0.49
				PVSM vs. NA	W = 3.066	0.002	r = 0.50
				PVSM vs. P2	W = −1.962	0.050	r = 0.32

**Table 4:** Test statistics (left) and multiple-comparison tests (right) for the two dependent variables RMSSD (top) and HF (bottom).

Main Effect	Test Statistics	*p*	Effect Size	Sig. Multiple Comp. Tests	Test Statistics	*p*	Effect Size
**RMSSD**	χ^2^[5] = 44.180	<0.001	W = 0.24	VSM vs. NA	W = 3.250	<0.001	r = 0.53
				VSM vs. P1	W = 3.740	0.023	r = 0.61
				VSM vs. RS	W = 2.453	0.014	r = 0.40
				NA vs. PVSM	W = 4.108	<0.001	r = 0.67
				NA vs. P2	W = 4.599	<0.001	r = 0.67
				P1 vs. PVSM	W = 4.599	<0.001	r = 0.75
				P1 vs. P2	W = 4.108	<0.001	r = 0.75
				RS vs. PVSM	W = −3.311	<0.001	r = 0.54
				RS vs. P2	W = −3.311	<0.001	r = 0.54

**HF**	χ^2^[5] = 37.263	<0.001	W = 0.20	VSM vs. P1	W = 3.740	<0.001	r = 0.61
				VSM vs. RS	W = 2.207	0.027	r = 0.36
				NA vs. PVSM	W = −2.942	0.003	r = 0.48
				NA vs. P2	W = −2.820	0.005	r = 0.46
				P1 vs. PVSM	W = −4.660	<0.001	r = 0.76
				P1 vs. P2	W = −4.782	<0.001	r = 0.78
				RS vs. PVSM	W = −3.250	0.002	r = 0.53
				RS vs. P2	W = −3.127	0.001	r = 0.51

**Table 5:** Results of the non-parametric correlations between the measures (dependent variables) of the experiment and the VSM-NA difference values of the 38 participants. Asterisks indicate significant correlations at *p* ≤ 0.05.

Measure	TBR	BAR	FAA	HR Mean	HRV Mean	LF/HF Ratio	SDNN	LF	RMSSD	HF
Rho [36]	0.02	0.13	−0.19	0.07	−0.04	−0.07	−0.34 *	−0.32 *	−0.17	−0.20
*p*	0.89	0.44	0.24	0.65	0.79	0.66	0.03	0.05	0.31	0.23

## Data Availability

The data presented in this study are available upon request from the corresponding author due to signed confidentiality agreements with each participant. Data may be obtained from the first author upon request.
